# “Mass gathering events and COVID-19 transmission in Borriana (Spain): A retrospective cohort study”

**DOI:** 10.1371/journal.pone.0256747

**Published:** 2021-08-26

**Authors:** Salvador Domènech-Montoliu, Maria Rosario Pac-Sa, Paula Vidal-Utrillas, Marta Latorre-Poveda, Alba Del Rio-González, Sara Ferrando-Rubert, Gema Ferrer-Abad, Manuel Sánchez-Urbano, Laura Aparisi-Esteve, Gema Badenes-Marques, Belén Cervera-Ferrer, Ursula Clerig-Arnau, Claudia Dols-Bernad, Maria Fontal-Carcel, Lorna Gomez-Lanas, David Jovani-Sales, Maria Carmen León-Domingo, Maria Dolores Llopico-Vilanova, Mercedes Moros-Blasco, Cristina Notari-Rodríguez, Raquel Ruíz-Puig, Sonia Valls-López, Alberto Arnedo-Pena

**Affiliations:** 1 Emergency Service, Hospital de la Plana, Vila-real, Castellon, Spain; 2 Public Health Center, Castelló de la Plana, Castellon, Spain; 3 Health Centers I and II, Borriana, Castellon, Spain; 4 Carinyena Health Center, Vila-real, Castellon, Spain; 5 Health Center, Onda, Castellon, Spain; 6 Health Center, La Vall d’Uixó, Castellon, Spain; 7 Villa Fátima School, Borriana, Castellon, Spain; 8 Department of Health Science, Public University Navarra, Pamplona, Spain; 9 Epidemiology and Public Health (CIBERESP), Madrid, Spain; Italian National Research Council (CNR), ITALY

## Abstract

**Objective:**

Mass gathering events (MGEs) are associated with the transmission of COVID-19. Between 6 and 10 March 2020, several MGEs related to the *Falles* festival took place in Borriana, a municipality in the province of Castellon (Spain). The aim of this study was to estimate the incidence of COVID-19 and its association with these MGEs, and to quantify the potential risk factors of its occurrence.

**Methods:**

During May and June 2020, a population-based retrospective cohort study was carried out by the Public Health Center of Castelló and the Hospital de la Plana in Vila-real. Participants were obtained from a representative sample of 1663 people with potential exposure at six MGEs. A questionnaire survey was carried out to obtain information about attendance at MGEs and COVID-19 disease. In addition, a serologic survey of antibodies against SARS-Cov-2 was implemented. Inverse probability weighted regression was used in the statistical analysis.

**Results:**

A total of 1338 subjects participated in the questionnaire survey (80.5%), 997 of whom undertook the serologic survey. Five hundred and seventy cases were observed with an attack rate (AR) of 42.6%; average age was 36 years, 62.3% were female, 536 cases were confirmed by laboratory tests, and 514 cases were found with SARS-CoV-2 total antibodies. Considering MGE exposure, AR was 39.2% (496/1264). A dose-response relationship was found between MGE attendance and the disease, (adjusted relative risk [aRR] = 4.11 95% confidence interval [CI]3.25–5.19). Two MGEs with a dinner and dance in the same building had higher risks. Associated risk factors with the incidence were older age, obesity, and upper and middle class versus lower class; current smoking was protective.

**Conclusions:**

The study suggests the significance of MGEs in the COVID-19 transmission that could explain the subsequent outbreak in Borriana.

## Introduction

Mass gathering events (MGEs) are important risk factors of severe acute respiratory coronavirus 2 (SARS-CoV-2) transmissions, which causes coronavirus disease 2019 (COVID-19) pandemic [1]. According to the World Health Organization [2] “Mass gatherings are events characterized by the concentration of people at a specific location for a specific purpose over a set period of time that have the potential to strain the planning and response resources of the host country or community”. MGEs cover different types of event and contexts such as public and private celebrations, festivals, religious events and pilgrimages, sporting and touristic events, and political meetings. The crucial role of MGEs in the global propagation of the disease has been evidenced in several countries, including China [3], Iran [4], Malaysia [5], Italy [6], Spain [7], France [8], Germany [9], Jordan [10], Malta [11], Switzerland [12], and Malawi [13]. Significant international efforts have been made to implement specific measures, risk assessment and surveillance, and event cancellations in order to prevent the spread of SARS-CoV-2 from MGEs [14–17]. The propagation of SARS-CoV-3 in these MGEs was measured with the basic reproductive number (Ro), which reflects the efficiency of transmission of the disease [18], and “is defined as the expected number of secondary cases produced by a single (typical) infection in a completely susceptible population” [19]. A meta-analysis of medical literature estimated a Ro = 3.38±1.40 from a range of 1.90–6.49 for the COVID-19 pandemic [20].

MGEs imply the gathering of people in restricted spaces, either indoor or outdoor, over a prolonged period of time, where food and/or drink are generally consumed, usually in close proximity to others, and involving the movement of populations [14, 21–23]. The conditions of MGEs have been associated with the spread of SARS-CoV-2, but few MGE studies [24, 25] have published quantification and adjustment for potential risk factors. In addition, several epidemiologic biases such as selection and misclassification have been observed in some studies of SARS-CoV-2 outbreaks, considering the novelty of the disease and the emergency situation [26].

Spain has had a high incidence of COVID-19 [27, 28], with a large number of COVID-19 outbreaks occurring in households, nursing homes, hospitals, workplaces and leisure facilities [29–33]. We studied a MGE COVID-19 outbreak that took place in the first wave of the pandemic. During March and April 2020 in Borriana, a municipality with 34,683 inhabitants located in the province of Castellon in the Valencian Community (Spain), a COVID-19 outbreak occurred with an incidence of 260 cases (749.6 cases per 100,000 inhabitants) confirmed by reverse-transcriptase polymerase chain reaction (RT-PCR) [34]. Between February and the first days of March 2020, before the COVID-19 outbreak in Borriana, several MGEs took place in connection with the traditional *Falles* festival, which is held annually in Borriana. Our hypothesis was that the MGEs held during the *Falles* festival were associated with the COVID-19 outbreak in Borriana.

Using a population-based retrospective cohort study, we aimed to estimate the association of the incidence of COVID-19 disease with the MGEs in Borriana, and quantify potential risk factors of its occurrence.

## Material and methods

### Description of MGEs during the *Falles* festival in Borriana

Borriana is a municipality located 5.7 km from the Mediterranean Sea in the province of Castellon, Spain. A series of MGEs took place between March 6 and 10, 2020 during the traditional *Falles* festival in Borriana. This popular festival is organized by people in the town’s different neighborhoods, clustered in social groups, known as a “*falla*” (singular) or “*falles*” (plural), with the purpose of bringing the festivities to the streets. *Falles* groups consider themselves as a large family. Their final objective is to build a monument with humorous scenes of daily life, which is burned on March 19 [35, 36]. During the 2020 festivities, 19 *falles* with a total of 2800 members were active in Borriana; each group had between 26 and 384 people and a median of 143 members. The members of the *falles* comprise around 8.1% of Borriana’s population.

The MGEs analyzed in this study took place in three locations: building A, purposely designed for MGEs with a surface area of 1670 m^2^ and a capacity of 900 people (three events); theater B (two events and a capacity of 884), and an outdoor square in the city of Valencia. Building A and theater B had air conditioning and ventilation equipment. The MGEs are described below:

First, a *pa-i-porta* (‘bring your own’ supper) (building A; March 6), a community dinner with an estimated total attendance of approximately 1400 people over a seven hour period (9:30 p.m. to 4:30 a.m.) and dancing after midnight.

Second, the Queen’s gala dinner (building A; March 7), with 400 people gathered together over six and a half hours (10:00 p.m. to 4:30 a.m.) and dancing after midnight. A detailed list of attendees and their distribution in building A was available.

Third, a trip to see fireworks in a square in Valencia (March 8). About 800 people gathered for half an hour (at 2:00 p.m.).

Fourth, a senior citizens’ dance (building A; March 8). Around 100 people attended for one and a half hours (5:30 p.m. to 7:00 p.m.).

Fifth, the theater awards gala (theater B; March 8): indoor show attended by 300 people for two hours (7:00 p.m. to 9:00 p.m.).

Sixth, the Queen’s offering (theater B; March 10): a show attended by 400 people for one and a half hours (10:30 p.m. to 0:00 a.m.).

A summary of the characteristics of these MGEs is presented in Table 1.

**Table 1:** Characteristics of Mass Gathering Events (MGEs) connected to the *Falles* festival in Borriana from March 6 to 10.

MGEs	Location	Date	Hours	People in attendance	Activities
*Pa-i-porta* supper	Building A	3/6/2020	7.00	1400^1^	Dinner and dance
Queen’s gala dinner	Building A	3/7/2020	6.30	400	Dinner and dance
Trip to Valencia	Square outdoor	3/8/2020	0.30	800	Attendance
Senior citizen’s dance	Building A	3/8/2002	1.30	100	Dance
Theater awards gala	Theater B	3/8/2020	2.00	300	Attendance
Queen’s offering	Theater B	3/10/2020	1.30	400	Attendance

1. Throughout the duration of the MGE.

### Design of the study

A population-based retrospective cohort study was carried out from May 14 to June 31, 2020 in Borriana. The study was jointly designed by the Public Health Center of Castellon and the Emergency Service of the Hospital de la Plana (HP) in Vila-real. The study had two phases: 1) a survey with a specific questionnaire to estimate the incidence of COVID-19, and 2) a serologic study of antibodies against SARS-CoV-2 to confirm cases with laboratory tests and to uncover the extent of the infection and the epidemic situation.

### Questionnaire survey

In the first phase, starting on May 14, 2020, a standardized questionnaire was administered to obtain information about MGE exposure, demographic characteristics, occupation, habits, health condition, symptoms of the disease, the evolution of COVID-19 disease, previously conducted COVID-19 laboratory tests, and family members affected by COVID-19. The study period covering COVID-19 cases ranged from January 1 to June 31, 2020. The questionnaire was completed through a telephone survey carried out by the health staff of HP, Borriana health centers, and other health centers in the Health Department of La Plana in Vila-real, Castellon. In the telephone survey, parents were requested to ask the questions to their children when a child had been chosen in the simple random sampling. In general, parents were able to answer the questionnaire. Social class was estimated from occupations; children’s social class was that of their parents. Two groups were considered: I and II included professional, managerial and technical occupations (upper and middle class); group III-VI included skilled, non-manual or manual, semi-skilled, and unskilled occupations (lower class).

Participants were recruited from two sources. First, a representative sample of the 19 *falles* (n = 2800 members) was obtained by simple random sampling with design effect 1 and 19 clusters (one per *falla)*, considering an attack rate of COVID-19 disease of 50% in MGEs, a confidence level of 80%, and an alpha error 5%. The sample comprised 1558 people, which corresponds to 82 people per *falla* for those with a population larger than 82, or all the members of the *falla* for those with a population smaller than 82. The Open-Epi program [37] was used to randomly select the participants from a numbered list of all members of each *falla* until 82 people, including adults and children, were obtained. The phone numbers of each selected participant or their family member were then obtained from the Borriana *Falles* organization. Second, in order to maximize the number of participants from the Queen’s gala dinner (n = 400), given that the complete list of attendees to the event was available, an additional random sample of 105 people from the list of diners was recruited. The same procedure described above was used to randomly select the 105 people.

### Serologic survey and laboratory tests

During the second phase, a serologic survey of anti-SARS-CoV-2 antibody prevalence was carried out from 23 to 27 June 2020 by the Clinical Analysis and Microbiology Service (CAMS) of HP. Qualitative detection of antibodies against SARS-CoV-2 was carried out by an electrochemiluminescence immunoassay (ECLIA) (Elecsys® Anti-SARS-CoV-2, Roche Diagnostics) [38]. IgG and IgM antibodies against SARS-CoV-2 were detected by a lateral flow immunochromatographic assay (LFIC) (Healgen Scientific LLC for COVID-19 IgG/IgM rapid test cassette) [39].

Additionally, information about previously conducted laboratory tests for COVID-19 was gathered during the questionnaire survey. These data consisted of 1) RT-PCR tests, including LightMix® Modular Sarbecovirus E-gene with the LightCycler® 480 II system (Roche, Basel, Switzerland) [40], 2) ECLIA with LFIC, and 3) rapid anti-SARS-CoV-2 antibody tests. These tests were conducted by HP and other public and private laboratories.

### Case definitions

A probable COVID-19 case was defined as a patient who presented clinical and epidemiological criteria of COVID-19 disease during the study period, according to the case-definition proposed by the Center for Disease Control and Prevention [41]. Clinical and epidemiological criteria must fulfill two conditions. The first is the reporting of at least two of the following symptoms: fever, chills, rigors, myalgia, headache, sore throat, and new olfactory and taste disorder; or one of the following symptoms: cough, shortness of breath, or difficulty breathing; or severe respiratory illness such as pneumonia or acute respiratory distress syndrome. The second condition is contact with a confirmed COVID-19 patient, or living in an area where a COVID-19 outbreak has been reported.

A confirmed COVID-19 case was defined as a patient who had positive antibodies of SARS-CoV-2 by ECLIA with LFIC, positive PCR, or positive rapid anti-SARS-CoV-2 antibody tests during the study period.

Non-cases (negative cases) were defined as participants who had no COVID-19 clinical criteria or had negative COVID-19 test results during the study period.

### Statistical methods

The program Epi-Info® version 7 [42] was used to calculate the sample size. COVID-19 attack rates (AR) were estimated as the quotient between cases and total exposed population, considering different variables. To estimate the risk of the MGEs, COVID-19 cases with onset of symptoms between March 6 and 31, 2020 were included. The *pa-i-porta* and Queen’s gala dinner MGE events were analyzed in greater detail than the other events, considering the attendance population and the potential risk of COVID-19. Chi2 and Fisher’s exact test were used in the comparisons among variables.

Associations of risk factors with COVID-19 were measured by the relative risk (RR) using Poisson regression and multilevel Poisson regression, considering *falles* as a level group with a 95% confidence interval (CI). In order to adjust for potential confounding factors, the directed acyclic graphs (DAGs) method [43] was used with the DAGitty® program (version 3.0) [44], together with inverse probability weighted regression [45] to obtain adjusted AR (aAR) and RR (aRR). The DAG provides a picture of the relationship between an exposure (mass gathering event) and an outcome (COVID-19 disease) and factors that could be potential confounders. An adjustment was made for these factors. The factors were age, sex, social class (upper and middle class versus lower class), chronic illness, family COVID-19 case and *falla* (social group); all these factors could alter the relationship between exposure and outcome. In addition, a sensitivity analysis was carried out including participants who were tested for COVID-19 disease, to gain more specificity of the results. The Stata ® program (version 14) [46] was used in the calculation.

### Ethical aspects

The study was approved by the director of the Public Health Center of Castellon and the management of the Health Department of La Plana, taking into account the situation of the COVID-19 pandemic in the province of Castellon. Participation was voluntary, and after an explanation of the study objective, oral informed consent was obtained from all participants (or their parents, in the case of minors) included in the study. To ensure explicit consent, several clarifications were made before administering the survey questionnaire, including voluntary participation, the option to withdraw from the study without prejudice, the opportunity to receive information about the study, a guarantee that personal information would be kept in the strictest confidentiality and that privacy would be safeguarded as the data collected would be used anonymously for scientific research; the researcher responsible for data collection was identified. When a child was selected in the sampling process, permission to participate was requested from their parents, who would also help them to answer the questionnaire; all the above clarifications were also made before the survey questionnaire was administered.

## Results

### Description of participants in the questionnaire survey

**Fig 1:**
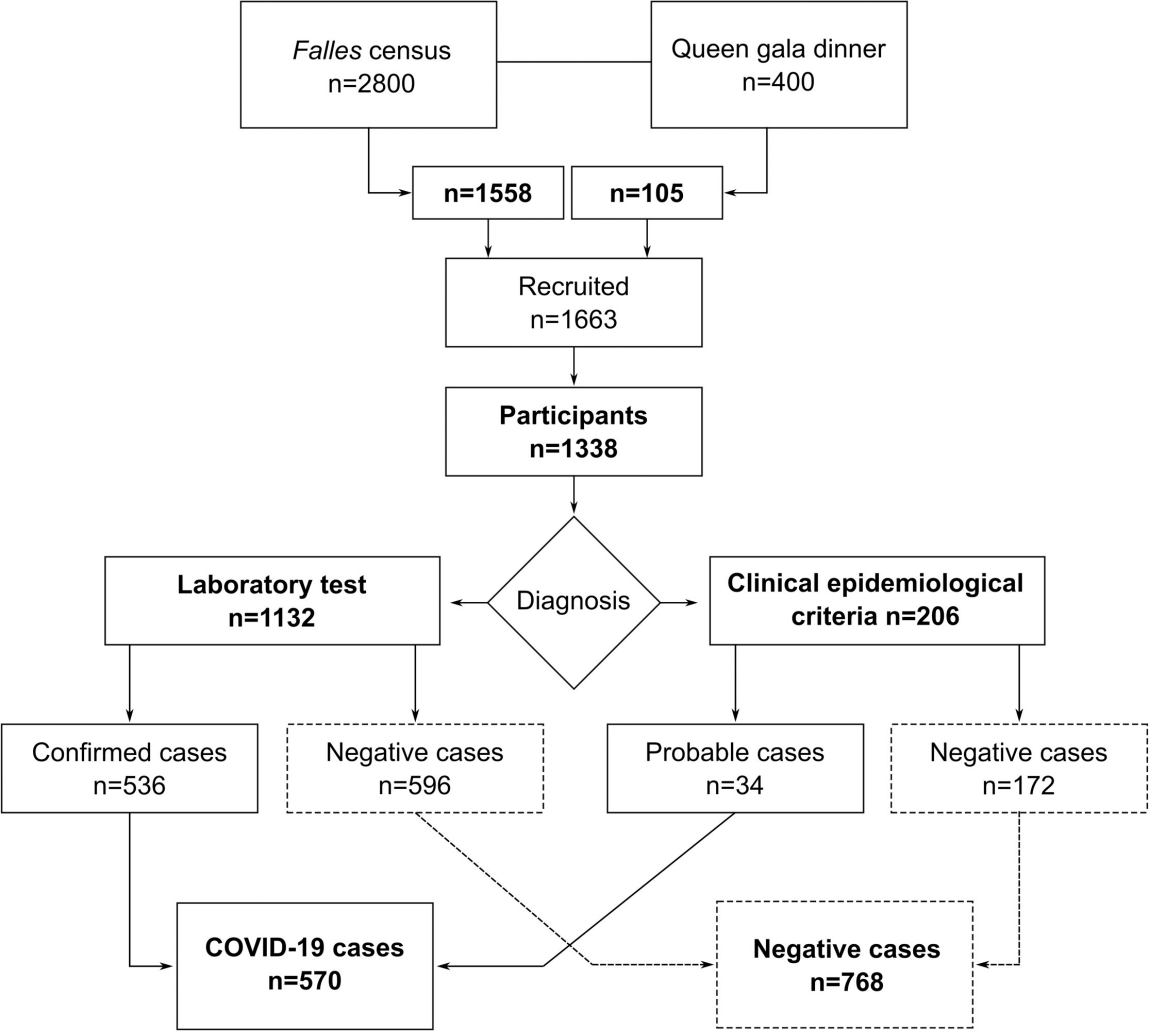
Flow diagram of the study population in the MGEs in Borriana between January and June 2020. COVID-19 cases included those diagnosed by laboratory tests (confirmed cases) or by clinical and epidemiological criteria (probable cases). Cases confirmed by laboratory tests included participants with positive results from the serologic survey as well as from private and public medical laboratories.

Participation rates in the questionnaire survey were 80.5% (1338/1663) (Fig 1). Participation was higher among females than males (58.9% versus 41.1%). The mean age was 33.9 ± 17.8 years (rank of 1–80 years) (Table 2). The most represented age groups were 45–64 years (28.2%) and 15–24 years (20.1%), and the least represented groups were 1–4 years (2.3%) and 65 years and above (3.4%) (Table 2). The occupation III-VI group (lower class) was higher than the I-II group (upper and middle classes) (74.3% versus 25.7%) (Table 2). Chronic illness was present in 30.9% of participants. The *pa-i-porta* event was the most highly attended MGE (60.5%), followed by the Queen’s gala dinner (24.8%). The theater award gala (12.4%) and the senior citizens’ dance (2.8%) had the lowest attendances (Table 2). A total of 397 participants in the questionnaire survey (25.3%) did not attend any MGEs.

**Table 2:** Characterization of the participants included in the study of Mass Gathering Events (MGEs) from January 1 to June 31, 2020, Borriana.

Variable	Participants (n)	Participants1 (%)	COVID-19 tests (n)	COVID-19 tests (%)
Total participants	1338	-	1132	84.61
Female	788	58.9	680	60.1
Male	550	41.1	452	39.9
Mean age ± SD2	33.9 ± 17.8	-	36.9 ± 17.1	-
Age (years)				
0–4	31	2.3	20	1.8
5–14	190	14.2	137	12.1
15–24	269	20.1	217	19.2
25–34	185	13.8	157	13.9
35–44	241	18.0	213	18.8
45–64	377	28.2	349	30.8
65 and above	45	3.4	39	3.4
Social class^3^^,^^4^				
Occupation I-II	339	25.7	283	25.0
Occupation III-VI	978	74.3	840	74.2
Chronic illness^5^				
Yes	411	30.9	364	32.2
No	920	69.1	763	67.4
MGE total attendees				
*Pa-i-porta* (n = 1400)	809 (57.8%)	60.5	740	65.4
Queen’s gala dinner (n = 400)	332 (83.0%)	24.8	317	28.0
Valencia trip (n = 800)	211 (26.4%)	15.8	190	16.8
Queen’s offering (n = 400)	239 (59.8%)	17.9	230	20.3
Senior citizens’ dance (n = 100)	38 (38.0%)	2.8	38	3.4
Theater awards gala (n = 300)	166 (55.3%)	12.4	151	13.3
No attendance	397	25.3	274	24.2

1. Of total participants.

2. Standard deviation.

3. Occupation group I and II: Professional, managerial and technical occupations (upper and middle class); Group III-VI: Skilled, non-manual or manual; semi-skilled; unskilled occupations (lower class).

4. Missing answer from 21 participants.

5. Missing answer from 7 participants.

### Description of participants in the serologic survey and COVID-19 laboratory tests

A total of 1132 participants (84.6%) underwent laboratory tests for COVID-19 disease (Table 2), considering both the tests carried out as part of the serologic survey (n = 997) and those from private and public medical laboratories (n = 135) (Fig 1, see Methods for further information). Participation rates in the serologic survey were 74.5% (997/1338). Female participation in laboratory tests for COVID-19 (including both the serologic survey and medical records) was higher (60.1%) than that of males (39.9%). The mean age of participants that underwent a COVID-19 laboratory test was 36.9 ± 17.1 years. The 45–64 years and 15–24 years age groups were the most highly represented (30.8% and 19.2%, respectively), while 0–4 years (1.8%) and 65 years and above (3.4%) were the least represented (Table 2). Participation of the occupation III-VI group (lower class) was higher than in the I-II group (upper and middle class) (74.2% versus 25.0%) (Table 2). A total of 32.2% of the participants reported suffering from a chronic illness (Table 2). MGE attendance among participants with COVID-19 laboratory tests showed the *pa-i-porta* event as the most highly attended event (65.4%), again followed by the Queen’s gala dinner (28.9%), while the theater award gala (13.3%) and the senior citizens’ dance (3.4%) had the lowest attendance (Table 2). A total of 274 participants (24.1%) with COVID-19 laboratory tests did not attend any MGEs (Table 2).

### Description of COVID-19 outbreak

During the study period, 570 COVID-19 cases were found (Fig 1, Table 2). Among these, 536 (94.0%) were confirmed cases with positive COVID-19 laboratory tests and 34 (6.0%) were probable cases as diagnosed according to clinical and epidemiological criteria (Fig 1). Specifically, confirmed cases included 514 participants (90.2%) with positive total anti-SARS-CoV-2 antibodies (32 of whom also reported a positive PCR test), seven participants (1.2%) with positive PCR only (a total of 39 participants had a positive PCR, 6.8%), and 15 participants (2.6%) with positive rapid anti-SARS-CoV-2 antibody tests. From the subset of 997 participants in the serologic survey, 508 were positive for anti-SARS-CoV-2 IgG antibodies, and eight participants were also positive for anti-SARS-CoV-2 IgM antibodies. AR was 42.6% (570/1338) among total participants, 47.3% (536/1132) among participants undergoing laboratory tests, and 16.5% (34/206) among those classified according to clinical and epidemiological criteria (Fig 1).

**Fig 2:**
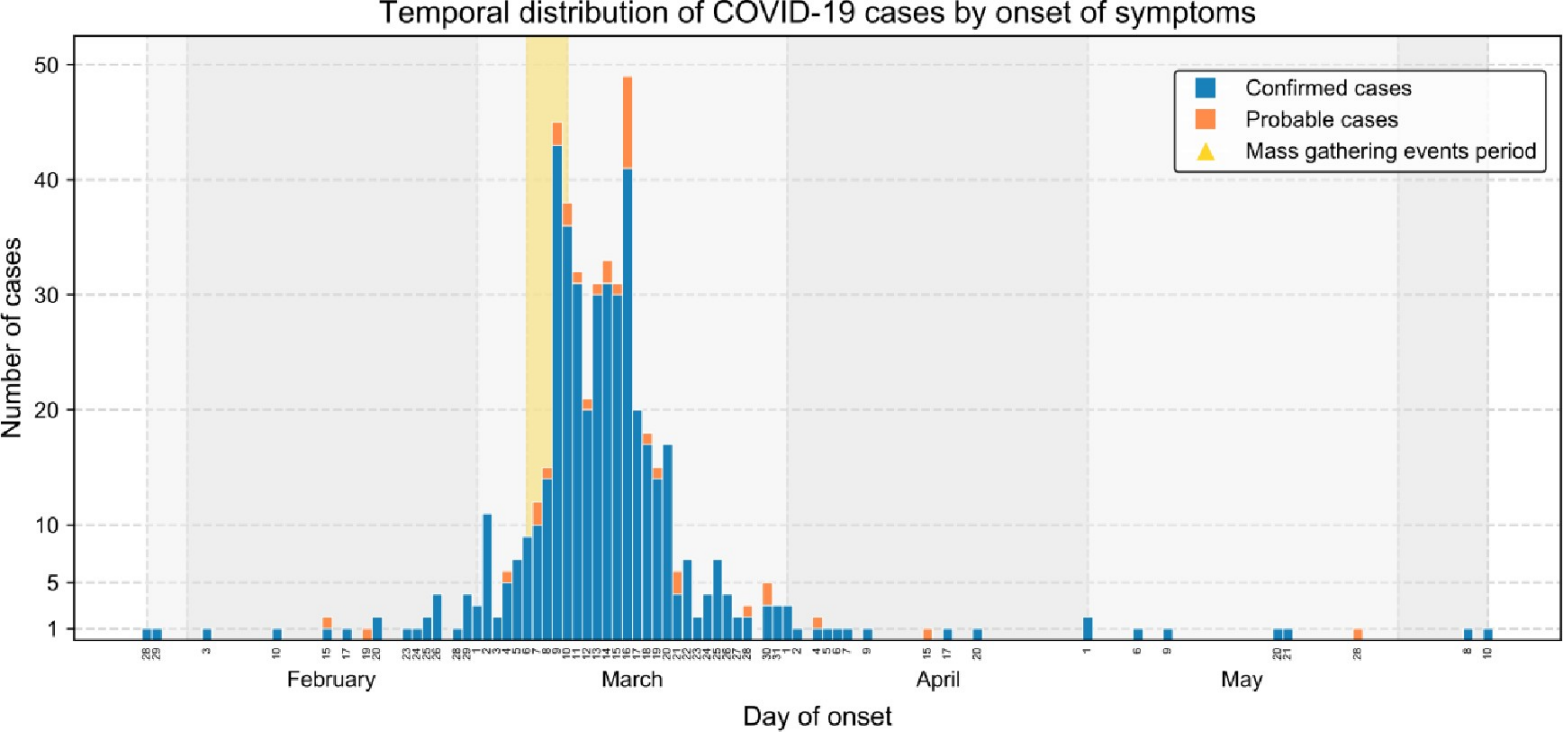
Temporal distribution of COVID-19 cases by onset of symptoms. The numbers of confirmed and probable COVID-19 cases are shown in blue and orange, respectively. The yellow vertical line highlights the time period when the six MGEs took place (March 6–10, 2020).

The temporal distribution of confirmed and probable COVID-19 cases is shown by onset of symptoms (Fig 2). The first cases were reported on 28 and 29 January 2020. The incidence then slowly progressed during February with a small peak of cases on March 2. Two maximum peaks of cases occurred on March 9 (45 cases) and March 16 (49 cases). After April, only isolated cases were reported, which is consistent with the general lockdown in Spain that was implemented on March 15. According to these observations, the distribution of COVID-19 cases in this study showed a bimodal epidemic curve with two maxima. Interestingly, the aforementioned peaks took place between 3 and 10 days after the MGEs (see Methods for further information). This indicates several point sources of the outbreak, with the *pa-i-porta* and Queen’s gala dinner events being the first ones on March 6 and 7, respectively.

#### Epidemiological characterization of COVID-19 cases

Regarding the sex of total cases, 355 (62.3%) were female and 215 (37.7%) were male (Table 3). The AR for males (39.1%) was smaller than for females (45.1%) (RR = 0.87, 95% CI 0.73–1.03) (Table 2). Similarly, COVID-19 incidence was lower in males than in females for both confirmed (44.9% versus 49.0%) and probable cases (12.2% versus 20.4%), although differences in AR were not significant.

**Table 3:** Characteristics of patients of COVID-19. From January 1 to June 31, 2020, Borriana.

Variables	Cases	Non-cases	Total	AR^1^ (%)	RR (95% IC)^2^	p-value
Total cases	570	768	1338	42.6	-	-
PCR positive	39	1299	1338	2.9	-	-
Hospitalizations	13	1325	1338	1.0	-	-
Deaths	1	1337	1338	0.07	-	´-
Sex						
Male	215	335	550	39.1	0.87(0.73–1.03)	0.110
Female	355	433	788	45.1	1.00	
Age mean ± SD^3^	36.4±17.1 32.0±18.0				1.01 (1.00–1.01)	0.001
Age groups (years)						
0–4	8	23	31	25.8	1.00	
5–14	58	133	190	30.5	1.11(0.56–2.44)	0.685
15–24	110	159	269	41.1	1.54(0.75–3.17)	0.243
25–34	72	113	185	38.9	1.48(0.71–3.08)	0.293
35–44	114	127	241	47.3	1.80(0.88–3.68)	0.110
45–64	189	188	377	50.1	1.92(0.94–3.90)	0.073
65 and above	19	26	45	42.2	1.56(0.68–3.57)	0.297
Confirmed cases						
Male	203	249	452	44.9	0.91(0.77–1.09)	0.322
Female	333	347	680	49.0	1.00	
Probable cases						
Male	12	86	98	12.2	0.63(0.30–1.28)	0.200
Female	22	86	108	20.4	1.00	
Asymptomatic						
Male	30	520	550	5.5	1.17(0.72–1.90)	0.519
Female	37	751	788	4.7	1.00	
Family with COVID-19^4^ Yes	347	254	601	57.7	1.91(1.59–2.23)	<0.001
No	221	506	727	30.4	1.00	
Medical assistance Yes	247	94	341	72.4	2.22(1.88–2.62)	<0.001
No	323	674	997	32.1	1.00	
Chronic illness^5^ Yes	189	222	411	46.0	1.13(0.95–1.35)	0.183
No	375	545	920	40.8	1.00	
Diabetes Mellitus^6^ Yes	10	18	28	35.7	0.84(0.45–1.56)	0.574
No	552	743	1295	42.6	1.00	
Cardiovascular diseases^6^Yes	69	66	135	51.1	1.23(0.95–1.58)	0.114
No	493	695	1188	41.5	1.00	
Hypertension^6^ Yes	47	51	98	48.0	1.14(0.84–1.54)	0.393
No	514	711	1225	42.0	1.00	
Hypothyroidism^7^ Yes	28	22	50	56.0	1.32(0.90–1.94)	0.150
No	534	740	1274	41.9	1.00	
Digestive diseases^6^ Yes	16	15	31	51.6	1.21(0.74–2.00)	0.450
No	545	747	1292	42.2	1.00	
Asthma^7^ Yes	11	27	38	29.0	0.66(0.36–1.20)	0.174
No	551	735	1286	42.8	1.00	
Allergic Rhinitis^7^ Yes	27	31	58	46.6	1.11(0.75–1.62)	0.613
No	535	731	1266	42.3	1.00	

1. AR = attack rate.

2. Adjusted for falla.

3. SD = Standard deviation.

4. Missing answers from 10 participants.

5. Missing answers from 7 participants.

6. Missing answers from 15 participants.

7. Missing answers from 14 participants.

Regarding age, cases were older than non-cases (36.4 ± 17.1 years versus 32.0 ± 18.0 years; p<0.001) (Table 3). When split by age groups, the highest ARs were found in the 55–64 years group (50.1%) and the 35–44 years group (47.3%), whereas the lowest ARs were observed in the 0–4 years group (25.8%) and 5–14 years group (30.5%) (Table 3), but no significant differences were observed among age groups when models were adjusted for *falla*.

Regarding the clinical presentation of the disease, a total of 503 cases (88.2%), including 469 confirmed cases and 34 probable cases, showed COVID-19 illness. The signs and symptoms reported by COVID-19 patients included weakness (56.3%), fever (55.1%), loss of smell and/or taste (53.8%), myalgia (51.3%), headache (46.2%) cough (49.0%), sore throat (35.7%), coryza (31.8%), diarrhea (26.6%), dermatologic lesions (12.1%), vomiting (5.7%) dyspnea (4.6%) and pneumonia (2.4%). The average disease duration was 16.1 ± 20.9 days, with a median of 7.0 days (rank 1–100). Long-term symptoms or aftermaths of COVID-19 were reported in 6.6% of cases. Of note, 247 cases (43.4%) sought medical assistance and 13 cases (2.3%) required hospitalization due to COVID-19. Only one death attributable to COVID-19 was reported during the period of the study. On the other hand, asymptomatic cases made up 12.5% of all confirmed cases (67/536), with an average age of 25.2 ± 17.6 years. No differences in the number of asymptomatic cases were found between males and females. Among total cases, 33.5% reported having a chronic disease, the most frequent of which were cardiovascular disease, hypertension, allergic rhinitis, and hypothyroidism (Table 3). No significant associations with COVID-19 incidence were found in any of the analyzed diseases. Finally, the effect of having a family member with COVID-19 at the time of disease onset was analyzed. AR among those participants who reported having a COVID-19 positive family member was 57.7% (347/601). In contrast, AR among participants who did not report a COVID-19 positive family member was 30.4% (221/727). Therefore, having a family member with COVID-19 increased the risk of COVID-19 incidence (RR = 1.91, 95% CI 1.59–2.23) (Table 3).

### Analysis of COVID-19 outbreak and MGEs

#### General analysis of MGEs

**Fig 3:**
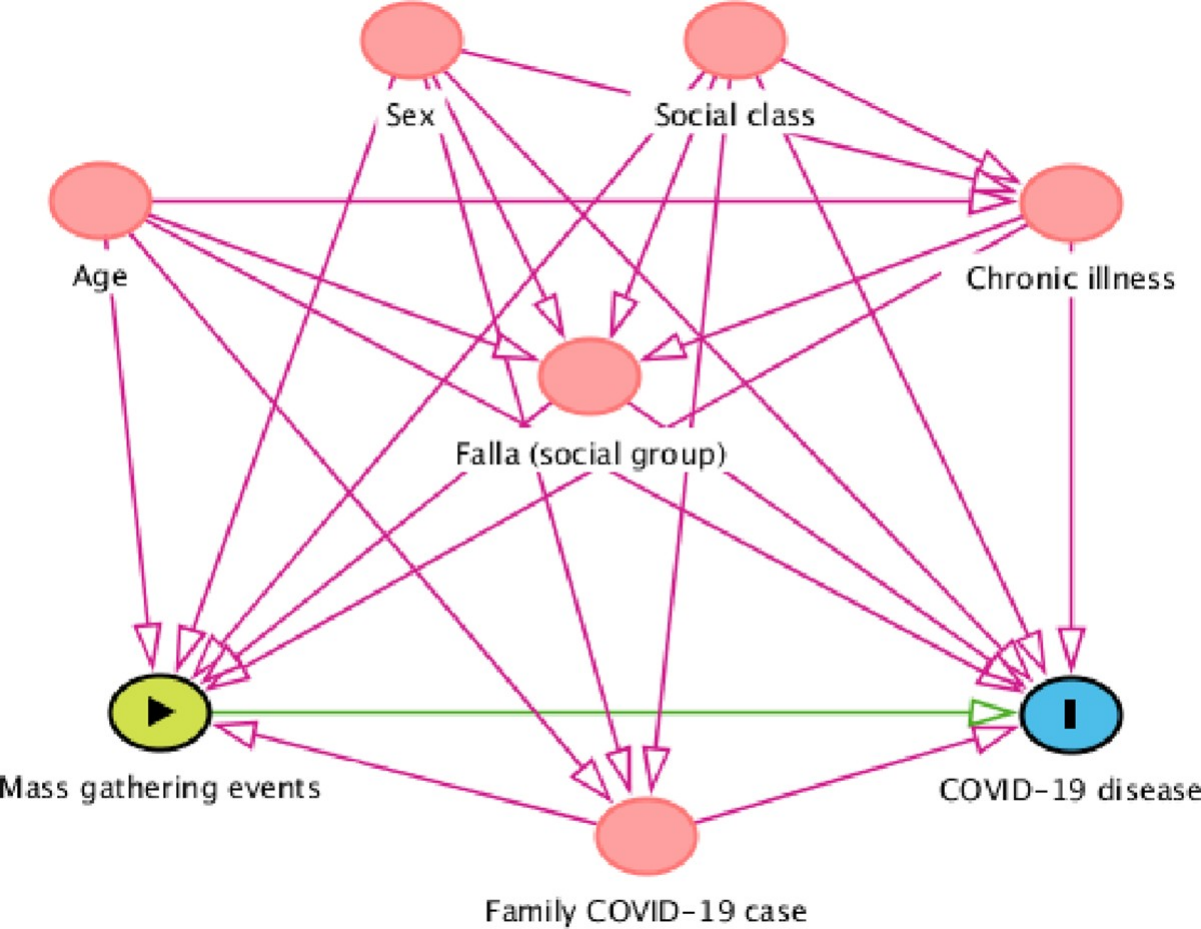
Directed Acyclic Graphs (DAG) of mass gathering events (exposure) effect on COVID-19 disease (outcome). Ancestors of exposure and outcome (in red). Based on DAGitty version 3.0.

Considering the dates of the MGEs (6 and 10 March 2020) and the temporal distribution of COVID-19 cases in the study (Fig 2), the analysis focused further on COVID-19 cases attributable to the MGEs associated with the *Falles* in Borriana. The 74 cases that presented the onset of symptoms or a positive test obtained before March 6 or after March 31 were therefore excluded since their illness onset (before the MGEs or more than three weeks after the last MGE) could not be related to the studied MGEs. Consequently, 1264 participants were included in the analysis with 496 cases, and AR of 39.2% (496/1264). DAGs were used to study MGEs (exposure) and COVID-19 disease (outcome) and potential confounding factors (Fig 3). Raw AR and adjusted (aAR), as well as RR and aRR, of MGEs are presented in Table 4. The aRR of males and females did not differ significantly. The aRR increased with age, from 0–4 years (aAR = 17.0%) to 35–44 years, which presented the highest values (aAR = 46.9%) (aRR = 2.77 95% CI 1.30–5.88). The age groups of 45–64 years and 65 years and above also presented high values (aAR = 44.2%) (aRR = 2.60 95% CI 1.22–5.52) and (aAR = 39.2%) (aRR = 2.31 95% CI 1.07–4.99), respectively. In addition, COVID-19 disease was associated with occupations I-II (upper and middle class) (aRR = 1.22 95% CI 1.06–1.40).

**Table 4:** Mass Gathering Event (MGE) attendance, Attack Rate (AR), adjusted AR (aAR), Relative Risk (RR), and adjusted (aRR). Confidence interval (CI). From March 6 to 31, 2020, Borriana.

Variables	Cases	Non-cases	AR (%)	RR (95% IC)	aAR^1^ (%)	aRR (95% IC)^1^	p-value
Male	189	335	36.1	0.87(0.73–1.04)	39.2	0.98(0.86–1.16)	0.785
Female	307	433	41.5	1.00	39.9	1.00	
Age 0–4 (years)^2^	6	23	20.7	1.00	17.0		
5–14	49	132	27.1	1.31(0.56–3.05)	25.6	1.51(0.69–3.31)	0.305
15–24	93	159	36.7	1.77(0.78–4.05)	35.9	2.12(0.99–4.54)	0.054
25–34	64	113	36.2	1.74(0.76–4.04)	38.1	2.25(1.04–4.83)	0.039
35–44	106	127	45.5	2.20(0.97–5.00)	46.9	2.77(1.30–5.88)	0.008
45–64	161	188	46.1	2.23(0.99–5.04)	44.2	2.60(1.22–5.52)	0.013
65 and above	18	26	40.9	1.97(0.78–4.98)	39.2	2.31(1.07–4.99)	0.032
Occupation I-II^1^	153	166	48.0	1.30(1.08–1.58)	45.7	1.22(1.06–1.40)	0.005
Occupation III-VI	340	585	36.8	1.00	37.6	1.00	
Chronic illness^1^ Yes	162	222	42.3	1.12(0.93–1.36)	39.7	1.01(0.88–1.17)	0.882
No	328	545	37.6	1.00	39.3	1.00	
Family with COVID-19 Yes	305	254	54.6	2.01 (1.67–2.41)	51.3	1.71(1.49–1.97)	<0.001
No	189	506	27.2		29.9	1.00	
Body Mass Index^1^^,^^3^	12	31	27.9	1.00	17.5	1.00	
<18.5 Kg/m^2^							
18.5–24.9 Kg/m^2^	203	327	38.3	1.32(0.96–1.80)	41.5	2.37(0.96–5.85)	0.062
25.0–29.9 Kg/m^2^	143	177	44.7	1.57(1.13–2.17)	45.2	2.58(1.04–6.40)	0.040
≥30.0 Kg/m^2^	81	74	52.3	1.68(1.18–2.40)	56.3	3.21(1.29–7.98)	0.012
Physical exercise^1^ ^3^ Yes	258	401	39.2	0.89(0.74–1.06)	40.1	0.88(0.77–1.01)	0.077
No	183	211	46.5	1.00	45.3	1.00	
Drink alcohol ^1^ ^3^ Yes	109	138	44.1	1.13(0.91–1.40)	46.9	1.14(0.99–1.32)	0.065
No	332	473	41.2	1.00	40.9	1.00	
Current smoker^1^ ^3^	75	197	27.6	0.66(0.51–0.85)	28.7	0.63(0.52–0.78)	<0.001
Ex smoker^1^ ^3^	108	100	51.9	1.25 (1.00–1.59)	50.8	1.13 (0.97–1.31)	0.122
Never smoker^1^ ^3^	256	311	45.9	1.00	45.0	1.00	
*Pa-i-porta*^2^ Yes	386	367	51.3	2.38(1.93–2.94)	49.9	2.15(1.80–2.56)	<0.001
No	110	401	21.5	1.00	23.4	1.00	
Queen’s gala dinner^2^ Yes	198	110	64.3	2.06(1.72–2.47)	61.9	1.90(1.67–2.17)	<0.001
No	298	658	31.2	1.00	32.5	1.00	
Valencia trip^2^ Yes	92	105	46.7	1.23(0.98–1.55)	47.9	1.25(1.06–1.46)	0.007
No	404	663	37.9	1.00	38.5	1.00	
Queen’s offering^2^ Yes	139	74	65.3	1.92(1.58–2.34)	62.0	1.77(1.54–2.04)	<0.001
No	357	694	34.0	1.00	35.0	1.00	
Senior citizens’ dance^2^ Yes	23	8	74.2	1.93(1.27–2.94)	57.3	1.48(1.14–1.93)	0.004
No	473	760	38.4	1.00	38.8	1.00	
Theater awards^2^ Yes	89	69	56.3	1.53(1.21–1.93)	54.1	1.44(1.22–1.70)	<0.001
No	407	699	36.8	1.00	37.6	1.00	
5 MGEs attended^2^	6	1	85.7	4.97(2.16–11.47)	77.7	4.11(3.25–5.19)	<0.001
4 MGEs attended^2^	52	18	74.3	4.31(3.00–6.20)	75.9	4.01(3.08–5.23)	<0.001
3 MGEs attended^2^	95	53	64.2	3.72(2.72–5.10)	61.2	3.24(2.49–4.21)	<0.001
2 MGEs attended^2^	125	116	51.9	3.01(2.23–4.06)	49.4	2.61(2.02–3.39)	<0.001
1 MGE attended^2^	152	263	36.6	2.13(1.59–2.84)	36.5	1.93(1.49–2.49)	<0.001
0 MGEs attended^2^	66	317	17.2	1.00	18.9	1.00	

1. Adjusted for age, sex, chronic illness, social class, family COVID-19 case, *falla*, and MGEs attended.

2. Adjusted for all factors except MGEs.

3. Age 15 years and above.

Those who had a family member with COVID-19 had a higher risk of SARS-CoV-2 infection (aRR = 1.71 95% CI 1.49–1.97). Additionally, the risk of contracting COVID-19 increased with higher body mass index (BMI), up to the group of 30 Kg/m^2^ or higher (aRR = 3.21 95% CI 1.29–7.98), and marginally with habitual alcoholic beverage consumption (aRR = 1.14 95% CI 0.99–1.32). Chronic illness and physical exercise were not associated with contracting COVID-19. On the other hand, the current smoker group presented lower risk (aRR = 0.63 95% CI 0.52–0.78). Regarding MGEs, the aAR ranged from 62.0% (the Queen’s offering) to 47.9% (the Valencia trip). The disease was associated with the *pa-i-porta* event with an aRR of 2.15 (95% CI 1.80–2.56), followed by the Queen’s gala dinner with aRR of 1.90 (95% CI 1.67–2.17). The other MGEs had lower aRR, but still presented a significant and considerable risk (Table 4). When MGE exposure was measured by the number of events the participants had attended, a dose-response relationship was found with raw and adjusted AR. The aAR for participants who attended no MGEs was 18.9%, increasing to 77.7% for participants who attended five MGEs (aRR = 4.11 95% CI 3.25–5.19).

#### Analysis of the *pa-i-porta* event

The analysis of the MGE with the highest number of participants, the *pa-i-porta*, together with different exposure variables, is shown in Table 5.

**Table 5:** Pa-i-porta, Mass Gathering Event (MGE) attendance, Attack Rate (AR), adjusted AR (aAR), Relative Risk (RR), and adjusted (aRR). Confidence interval (CI). From March 6 to 31, 2020, Borriana.

Variables	Cases	Non- cases	AR (%)	RR (95% IC)	aAR (%)	aRR(95% IC)^1^	p-value
Total	386	367	51.3				
Male	143	132	52.0	1.02(0.83–1.26)	49.3	0.96(0.83–1.11)	0.407
Female	243	235	50.8	1.00	51.5		
Attendance time^2^							
Less than half	32	37	46.4	1.00	44.2	1.0	
Half	111	143	43.7	0.94(0.64–1.40)	44.5	1.01(0.75–1.35)	0.956
More than half	104	86	54.7	1.18(0.80–1.75)	53.6	1.21(0.91–1.62)	0.189
All the time	138	100	58.0	1.25(0.85–1.84)	58.4	1.32(0.98–1.74)	0.051
Dinner^2^							
Ate own food	67	63	51.5	0.97(0.74–1.27)	55.5	1.07(0.88–1.29)	0.505
Ate *falla* food	268	238	53.0	1.00	52.0	1.00	
Ate other food^2^							
Yes	110	92	54.5	1.08(0.86–1.36)	51.0	1.00(0.85–1.18)	0.839
No	225	222	50.3	1.00	50.8	1.00	
Cake consumption^2^							
Yes	218	182	54.5	1.14 (0.99–1.43)	53.2	1.10(0.94–1.28)	0.236
No	120	132	47.6	1.00	48.7	1.00	
Drank alcohol beverages at this event^2^ ^3^							
Yes	251	217	53.6	0.95(0.74–1.21)	54.0	0.98(0.83–1.16)	0.913
No	87	67	56.5	1.00	54.9	1.00	
Attendance^2^							
Only dance	43	58	42.6	0.81(0.59–1.11)	44.2	0.84 (0.67–1.06)	0.137
All event	332	300	52.5	1.00	52.5	1.00	
Dance^2^							
Not at all	94	93	51.3	1.00	47.6	1.00	
A little	104	100	51.0	1.01(0.77–1.34)	48.5	1.04(0.84–1.29)	0.708
Some of the time	103	97	51.5	1.02(0.77–1.35)	53.2	1.12(0.91–1.38)	0.292
A lot	82	71	53.6	1.06(0.79–1.43)	56.8	1.19(0.96–1.48)	0.120
Dinner table with a COVID-19 symptomatic case^4^							
Yes	338	311	52.4	1.18(0.86–1.62)	55.7	2.19(1.40–3.42)	0.001
No	44	56	44.0	1.00	25.5		
Quadrant of building A^5^							
Upper left	130	118	52.1	1.36 (1.01–1.84)	47.0	1.10 (0.79–1.52)	0.570
Lower left	118	113	51.1	1.32 (0.98–1.81)	51.7	1.18 (0.82–1.16)	0.309
Upper right	71	35	67.0	1.74 (1.24–2.45)	67.7	1.44 (1.12–1.85)	0.005
Lower right	63	101	38.4	1.00	40.0	1.00	

1. Adjusted for age, sex, chronic illness, social class, family COVID-19 case, and *falla*.

2. Missing information of two cases.

3. Age 15 years and above.

4. Adjusted age, sex, mean of attendance time, and mean MGEs attended.

5. Adjusted for median of MGEs attended.

The aRR by sex was similar in females and males. Interestingly, COVID-19 disease was marginally associated with the total time attended in a dose-response relationship from less than half of the event up to the whole event (aRR = 1.32 95% CI 0.98–1.74). The food or alcoholic beverages provided by the event organizers, or those brought to the event by the participants (which included cakes or other foods) were not associated with the disease. Participants who did not participate in the dinner and only attended the dance had a lower risk, but with limited effect (aRR = 0.84 95% CI 0.67–1.16). In addition, more time spent dancing was a marginal risk factor (aRR = 1.19 95% CI 0.96–1.48). Two groups of participants were defined according to whether or not they shared a dinner table with a participant reporting onset of COVID-19 symptoms between February 26 and March 6. The exposed group presented a higher aAR of disease than those who were not exposed, 55.7% versus 25.5% (aRR = 2.19 95% CI 1.40–3.42). Finally, the location and distribution of the dinner tables in building A were analyzed. To this end, the dinner tables were classified into four quadrants and an adjusted analysis was carried out. Results showed that the upper right quadrant had a higher COVID-19 incidence than the lower right quadrant (aRR = 1.44 95% 1.12–1.85), but no difference was found with the other quadrants (Table 5).

#### Analysis of the Queen’s gala dinner event

The Queen’s gala dinner was the MGE with the second highest number of participants in the study (Table 6). The aRR by sex was similar in females and males. The time of attendance at this event presented a dose-response relationship from less than half attendance to whole event attendance (aRR = 1.76 95% CI 1.18–2.62). At dinner, several dishes were consumed: starters (tomato bread, pork sausages, cheese and *foie gras* salad and scallops), second course (sirloin), and dessert (chocolate cream). The consumption of these foods and alcoholic beverages was not associated with COVID-19 risk. Next, two groups of participants were defined according to whether or not they shared a dining table with a participant with COVID-19 disease and symptoms onset between February 27 and March 7. The exposed group showed a higher aAR than the non-exposed group (76.1% and 58.6%, respectively) (aRR = 1.30 95%1.10–1.53). The adjusted analysis of the incidence of COVID-19 cases across the four quadrants of tables in building A (same building as for the *pa-i-porta* event), revealed that the lower left quadrant had the highest COVID-19 incidence and differed from that of the lower right quadrant, which was the one with the lowest incidence (aRR = 1.29 95% 1.00–1.65). No differences were found with the other table quadrants (Table 6). Interestingly, the lower right quadrant was the one with the lowest COVID-19 incidence in both the *pa-i-porta* and the Queen’s gala dinner events.

**Table 6:** Queen’s gala dinner, Mass Gathering Event (MGE) attendance, Attack Rate (AR), adjusted AR (aAR), Relative Risk (RR), and adjusted (aRR). Confidence interval (CI). From March 6 to 31, 2020, Borriana.

Variable	Cases	Non-cases	AR (%)	RR (95% IC)	aAR (%)	aRR(95% IC)^1^	p-value
Total	198	110	64.3				
Male	75	44	63.0	0.97(0.72–1.29)	62.1	0.96(0.80–1.15)	0.653
Female	123	66	65.1	1.00	64.7	1.00	
Attendance time							
Less than half	11	13	45.8	1.00	39.8	1.00	
Half	31	16	66.0	1.44 (0.78–2.88)	65.3	1.64(1.05–2.55)	0.028
More than half	42	31	57.5	1.26(0.64–2.44)	56.3	1.42(0.91–2.20)	0.121
All time	114	50	69.5	1.52(0,82.2.82)	70.2	1.76(1.18–2.62)	0.005
Dinner							
Tomato bread Yes	185	101	64.7	0.94(0.51–1.73)	65.0	1.14(0.89–1.46)	0.312
No	11	5	68.8	1.00	57.1	1.00	
Pork sausages Yes	182	99	64.8	0.97(0.56–1.67)	64.7	0.88(0.71–1.09)	0.249
No	14	7	66.7		73.6	1.00	
Foie salad Yes	163	93	63.7	0.89(0.61–1.29)	63.9	0.82(0.68–0.97)	0.022
No	33	13	71.7		78.4	1.00	
Scallops Yes	158	86	64.8	0.99(0.69–1.41)	65.3	0.96(0.79–1.17)	0.673
No	38	20	63.8		68.1	1.00	
Sirloin Yes	173	96	64.3	0.92(0.60–1.43)	65.0	0.77(0.67–0.89)	<0.001
No	23	10	69.7	1.00	84.4	1.00	
Chocolate Yes	165	82	66.8	1.19(0.81–1.74)	67.5	1.13(0.91–1.40)	0.263
No	31	24	54.5		59.8	1.00	
Drank alcohol ^2^ Yes	163	94	63.4	0.93(0.65–1.35)	63.6	1.20(0.95–1.52)	0.123
No	34	14	70.8		52.8	1.00	
Dinner table with a COVID-19 symptomatic case^3^							
Yes	88	35	71.5	1.18(0.89–1.57)	76.1	1.30(1.10–1.53)	0.002
No	103	67	60.6	1.00	58.6	1.00	
Quadrant of building A^4^							
Upper left	44	18	71.0	1.27 (0.84–1.91)	63.4	0.98(0.72–1.34)	0.917
Lower left	43	15	74.1	1.33 (0.88–2.00)	83.0	1.29(1.00–1.65)	0.047
Upper right	57	32	64.0	1.44 (0.78–1.68)	64.6	1.00(0.76–1.32)	0.990
Lower right	47	37	56.0	1.00	64.4	1.00	

1. Adjusted for age, sex, chronic illness, social class, family COVID-19 case, and *falla*.

2. Age 15 years and above.

3. Adjusted for age, sex, chronic illness, social class, family COVID-19 case, *falla*, attendance time, quadrant, and number of MGEs attended.

4. Adjusted for age, sex, chronic illness, social class, family COVID-19 case, *falla*, symptomatic case in dinner table, and number of MGEs attended.

Finally, our analysis also found that four out of the five food handlers who served in the two MGE dinners were confirmed COVID-19 cases by laboratory tests. Three of them reported having COVID-19 symptomatology starting on March 16, 17, and 24 March 2020, respectively, after the MGEs were over. One case was asymptomatic.

#### Sensitivity analysis

The sensitivity analysis included the 1064 participants with laboratory tests of COVID-19 and cases between 6 and 31 March 2020 (Tables 7 and 8). The AR of this group was 44.0% from 468 COVID-19 cases and 596 non-cases. Some variations with the previous findings were found, considering its lower sample size and the higher age of participants (Table 2). Risk factors such as age and BMI lost significance, but alcohol consumption was associated with COVID-19 disease. Other factors did not show considerable changes. The *pa-i-porta* and the Queen’s gala dinner maintained significant associations with COVID-19, including attendance time, table shared with a COVID-19 symptomatic participant, and quadrants in building A. In the Queen’s gala dinner, quadrants lost significance.

**Table 7:** Sensitivity analysis. Participants with laboratory tests of SARS-CoV-2 and mass gathering event (MGEs) attendance, attack rate (AR) adjusted AR (aAR), and adjusted (aRR). Confidence interval (CI). From March 6 to 31, 2020, Borriana.

Variables	Cases	Non-cases	AR (%)	aAR (%)	aRR (95% IC)^1^	p-value
Male	180	249	42.0	44.1	1.01(0.89–1.16)	0.862
Female	288	347	45.4	43.6	1.00	
Age 0–4 (years)^2^	4	14	22.2	16.4	1.00	
5–14	44	84	34.4	29.4	1.79(0.50–6.38)	0.370
15–24	88	115	43.4	42.1	2.46(0.69–9.00)	0.143
25–34	60	90	40.0	42.3	2.58(0.73–9.08)	0.142
35–44	100	105	48.8	49.9	3.03(0.87–10.63)	0.083
45–64	155	167	48.1	46.7	2.84(0.81–9.94)	0.102
65 and above	17	21	44.7	41.1	2.50(0.71–8.83)	0.154
Occupation I-II^1^	142	122	53.8	51.6	1.23(1.07–1.42)	0.003
Occupation III-VI	323	469	40.8	41.9	1.00	
Chronic illness^1^ Yes	155	184	42.9	43.7	0.99(0.87–1.15)	0.992
No	309	411	45.7	43.9	1.00	
Family with COVID-19 Yes	293	213	57.9	55.6	1.64(1.43–1.88)	<0.001
No	174	376	31.6	33.9	1.00	
Body Mass Index^1^^,^^3^ <18.5 Kg/m^2^	11	24	31.4	6.7	1.00	
18.5–24.9 Kg/m^2^	189	252	42.9	45.6	6.81(0.41–113.91)	0.182
25.0–29.9 Kg/m^2^	139	154	47.4	48.8	7.29(0.44–121.98)	0.167
≥30.0 Kg/m^2^	79	66	54.5	59.1	8.83(0.53–401.56)	0.130
Physical exercise^1^^,^^3^ Yes	244	322	43.1	44.1	0.90(0.78–1.02)	0.109
No	176	176	50.0	49.2	1.00	
Drank alcohol ^1^^,^^3^ Yes	104	108	49.1	53.4	1.19(1.04–1.37)	0.015
No	316	390	44.8	44.8	1.00	
Current smoker^1^^,^^3^	68	169	28.7	30.7	0.62(0.50–0.75)	<0.001
Ex smoker^1^^,^^3^	104	83	55.6	54.8	1.10 (0.95–1.28)	0.215
Never smoker^1^^,^^3^	246	245	50.1	49.8	1.00	
5 MGEs attended^2^	6	1	85.7	82.6	3.62(2.83–4.61)	<0.001
4 MGEs attended^2^	52	15	77.6	78.7	3.44(2.63–4.49)	<0.001
3 MGEs attended^2^	94	48	66.2	64.3	2.81(2.15–3.67)	<0.001
2 MGEs attended^2^	121	101	54.5	53.0	2.32(1.78–3.02)	<0.001
1 MGE attended^2^	139	224	38.3	37.9	1.66(1.27–2.16)	<0.001
0 MGEs attended^2^	56	207	21.3	22.9	1.00	

1. Adjusted for age, sex, chronic illness, social class, family COVID-19 case, *falla*, and MGEs attended.

2. Adjusted for all factors except MGEs.

3. Age 15 years and above.

**Table 8:** Sensitivity analysis. Participants with laboratory tests of SARS-CoV-2 and the pa-i-porta and Queen’s gala dinner mass gathering event (MGE) attendance, attack rate (AR), adjusted AR (aAR), and adjusted (aRR). Confidence interval (CI). From March 6 to 31, 2020, Borriana.

Variables	Cases	Non-cases	AR (%)	aAR (%)	aRR (95%IC)^1^	p-value
*Pa-i-porta*						
Attendance^1^ Yes	371	316	54.0	53.0	1.96(1.64–2.35)	<0.001
No	97	280	25.7	27.0	1.00	
Attendance time^1^ ^2^						
Less than half	30	33	47.6	44.6	1.00	
Half	102	119	46.2	45.6	1.02(0.75–1.38)	0.887
More than half	102	74	58.0	52.3	1.26(0.94–1.70)	0.122
All the time	136	89	60.4	61.2	1.37(1.03–1.83)	0.031
Dinner table with a COVID-19 symptomatic case^3^						
Yes	326	263	55.3	58.8	2.57(1.45–4.83)	0.001
No	41	46	47.1	22.9	1.00	
Quadrants building A^4^						
Upper left	127	93	57.7	51.8	1.25(0.88–1.77)	0.212
Lower left	112	98	53.3	53.3	1.29(0.95–1.74)	0.105
Upper right	69	31	69.0	69.3	1.67(1.34–2.08)	0.000
Lower right	59	87	40.4	41.5	1.00	
Queen’s gala dinner						
Attendance^1^ Yes	196	98	66.7	65.5	1.80(1.58–2.05)	<0.001
No	272	498	35.3	36.4	1.00	
Attendance time^1^						
Less than half	11	11	50.0	42.4	1.00	
Half	31	14	68.9	69.9	1.65(1.08–2.51)	0.019
More than half	42	26	61.8	61.0	1.44(0.94–2.18)	0.086
All the time	112	47	70.4	71.0	1.67 (1.15–2.45)	0.008
Dinner table with a COVID-19 symptomatic case^5^						
Yes	88	31	74.0	74.7	1.19 (1.01–1.40)	0.037
No	101	61	62.4	62.8	1.00	
Quadrant of building A^6^						
Upper left	43	18	70.5	66.9	1.01(0.73–1.35)	0.971
Lower left	43	14	75.4	84.9	1.26(0.98–1.63)	0.075
Upper right	56	26	68.3	63.8	0.95(0.71–1.27)	0.718
Lower right	47	34	58.0	67.2	1.00	

1. Adjusted for age, sex, chronic illness, social class, family COVID-19 case, and *falla*.

2. Missing information in two cases.

3. Adjusted age, sex, median of attendance time, and median MGEs attended.

4. Adjusted for median of MGEs attended.

5. Adjusted for age, sex, chronic illness, social class, family COVID-19 case, *falla*, attendance time, quadrant, and number of MGEs attended.

6. Adjusted for age, sex, chronic illness, social class, family COVID-19 case, *falla*, symptomatic case in dinner table, and number of MGEs attended.

## Discussion

The results of this study indicate a high transmission of COVID-19 disease in the MGEs studied, which may be considered as a community outbreak with several point sources from March 6 to 10 [47]. Respiratory transmission was the predominant mode of propagation by droplets from patients to exposed persons; contact via fomites was less frequent [48, 49]. Airborne transmission has been suggested in relation to COVID-19 outbreaks in different places, and is a plausible route [50–52]. The two MGEs with the highest incidence of the disease included a dinner, as in other COVID-19 outbreaks, but food-borne transmission can be discarded in this study [53, 54].

Some characteristics of this MEG outbreak may be highlighted, including its magnitude and impact in the population of Borriana, the diversity of places where it occurred indoors (building and theater) and outdoors (square), the higher risk of COVID-19 associated with the number of MGEs attended, the estimation of risk for attendance at no MGEs, the detection of risk factors (obesity, old age, and upper and middle class versus lower class) with current smoking as a protective factor, and the rapid spread with high ARs.

In the study, the AR was 42.6%, and during the period of the MGEs, 39.2% with 94.0% of confirmed cases. If the AR were extrapolated to *falles* members, the total of COVID-19 cases could be between 1193 (95% CI 1269–1117) and 1098 (95% CI 1173–1023), respectively. In addition, a high proportion of cases had family members with COVID-19, suggesting a high secondary transmission among families, in line with Thompson and co-authors and with a study of secondary attack rate of COVID-19 infections in Castellon [55, 56]. It may explain the high incidence in the municipality of Borriana, which was the highest in the province of Castellon during the first outbreak period. Considering the period February to July 2020, the incidence of COVID-19 in Borriana was 307 cases with positive PCR results (885 per 100,000 inhabitants) and 40 deaths (1.2 per 1000 inhabitants) [34]. As a comparison, the COVID-19 incidence in Castellon de la Plana, the provincial capital (170,000 inhabitants), was 463 cases (272.4 per 100,000 inhabitants) and 50 deaths (0.29 per 1,000 inhabitants). The high mortality in Borriana is striking [57, 58], and it may be related to the prevalence of cardiovascular risk factors in this municipality [59]. Regarding the incidence of COVID-19, the study found 536 confirmed cases, 39 cases with positive PCR results; these were the only cases reported by the health authorities. It may be estimated that for every reported case, there could have been around 13 or 14 cases, implying that 92.7% of cases were undiagnosed. This situation is in line with two studies on undocumented infections of SARS-CoV-2 in China (86% and 93%), and France (86%), respectively [60–62].

In the context of COVID-19 outbreaks related to MGEs, different models have been proposed to estimate the number of cases deriving from MGEs or Ro values [63, 64]. Apart from assuming several premises, these models need a constant infection rate or Ro. As a tentative approximation and using the classic SIR (susceptible, infected, recovered) infection process model [65], we explored our results to obtain the Ro from the infection rate based on the date of onset of cases, number of contacts, and duration of infectiousness. We used the formula [66]:

Ro=PIxCRxDI

where PI = “average probability a contact will be infected over duration of a relationship”; CR = “average rate of getting into contact”; and DI = “average duration infectiousness”. We defined PI, the infection rate, as new cases of COVID-19 divided by the susceptible participants per day; CR as a number of potential contacts, following the study of Tupper and co-authors [63], and DI as 10 days duration of infectiousness [67]. We considered a period of 19 days from the first MGE to the last MGE, plus a 14-day maximum incubation period for COVID-19. The results are shown in Table 9.

**Table 9:** Estimation of the basic reproductive number (Ro) from the MGE COVID-19 outbreak between 6 and 10 March 2020 plus 14 days.

Days/Date infection onset	New cases (a)	Total cases (I) (b)	Susceptible (S) (c)	Infection rate (a/c)	Basic reproduction number Ro^1^ Numbers of contacts^2^		
					5	10	15
3/6/2020: 0^3^	9	9	1264	0.00712	0.36	0.71	1.07
3/7/2020: 1^3^	11	20	1255	0.00877	0.44	0.88	1.32
3/8/2020: 2^3^	15	35	1244	0.01206	0.60	1.21	1.81
3/9/2020: 3	45	80	1229	0.03662	1.83	3.66	5.49
3/10/2020^3^ 4	39	119	1184	0.03294	1.65	3.29	4.94
3/11/2020: 5	32	151	1145	0.02795	1.40	2.80	4.19
3/12/2020: 6	21	172	1113	0.01887	0.94	1.89	2.83
3/13/2020: 7	31	203	1092	0.02839	1.42	1.84	4.26
3/14/2020: 8	33	236	1061	0.03110	1.56	3.11	4.67
3/15/2020: 9	30	266	1028	0.02918	1.46	2.92	4.38
3/16/2020:10	48	314	998	0.04810	2.41	4.81	7.22
3/17/2020:11	20	334	950	0.02105	1.03	2.05	3.08
3/18/2020:12	18	352	930	0.01935	0.97	1.94	2.90
3/19/2020:13	15	369	912	0.01644	0.82	1,64	2.47
3/20/2020:14	15	382	897	0.01672	0.84	1.67	2.51
3/21/2020:15	6	388	882	0.00680	0.34	0.68	1.02
3/22/2020:16	7	395	876	0.00799	0.40	0.80	1.20
3/23/2020:17	2	397	869	0.00230	0.12	0.23	0.35
3/24/2020:18	4	401	867	0.00461	0.23	0.46	0.69
3/25/2020:19	7	408	863	0.00811	0.41	0.81	1.22

1. Ro = infection rate x contact numbers x duration infectiousness (10 days).

2. Considering average numbers of contacts in conferences, lectures, and restaurants (5, 10, and 15).

3. Date with MGE.

Considering the infection rate per day, an increase was observed from 0.00172 on the first day to 0.04810 on March 16 and a decline until 0.00811 on March 25. The countrywide lockdown began on March 15 and has been shown to have had a high effect [68]. With five contacts, Ro>1.0 reached only 8 days and a maximum of 2.41; with 10 contacts, Ro>1.0 reached 13 days and a maximum of 4.81; and with 15 contacts, Ro>1.0 reached 19 days and a maximum of 7.22 on the 16th day. The decline of the Ro was very steep from the maximum and Ro<1.0 values were observed, indicating that the epidemic would soon decline [69]. These results reflect the high transmission of the disease associated with MGEs.

To explore the expected numbers of cases in the COVID-19 outbreak associated with the MGEs, we followed the approach of Tupper and co-authors [63], considering the probability of infection (β), the duration time (T), time of contact (τ) and the number of contacts (k). We calculated k from two MGEs (*pa-i-porta* and the Queen’s gala dinner). The formula yields the expected number of new infections:

$$R_{event}=(kT/\tau )(1‐e^{\beta \tau })$$

Attendances at the *pa-i-porta* and Queen’s gala dinner events were used to estimate expected cases and compare them with observed cases. For the *pa-i-porta* event, the number of contacts was 85 for less than half the attendance time and 60 for more than half the attendance time, to obtain a similar number of observed and expected cases. For the Queen’s gala dinner the numbers of contacts were 20 for less than half the attendance time and 40 for more than half the attendance time. Considering that 9 and 11 cases of COVID-19 (Table 10) had the onset of symptoms the day of the *pa-i-porta* and the Queen’s gala dinner, respectively, the number of contacts k could be divided by 9 and 11, and contacts could be 7 to 10 for *pa-i-porta* and 2 to 4 for the Queen’s gala dinner. These results could explain the spread of the epidemic, and underline the importance of the duration of the event, the number of contacts, and the number of participants, which were much higher in the *pa-i-porta* than in the Queen’s gala dinner. However, a major limitation of this approach is the theoretical assumption of homogeneous mixing of population in the epidemic [70]. In addition, asymptomatic or undiagnosed cases may bias the results and 67 cases were asymptomatic in our study.

**Table 10:** Attendance at *pa-i-porta* and Queen’s gala dinner MGEs and number of contacts (k) to obtain expected cases of COVID-19 compared with observed cases from the formula of Tupper and co-authors [63].

MGEs	Attendance time	Probability infection β	Time T hours	Contact time τ	Number contacts k	Number expected Cases	Number observed cases
*Pa-i-porta*	Less than half	0.465	3.30	0.2	85	132.1	132
	More than half	0.594	7.0	0.2	60	235.2	238
Queen’s gala dinner	Less than half	0.627	3.15	0.2	20	41.2	42
	More than half	0.678	6.30	0.2	40	152.2	154

1. Infection rates of attendance at *pa-i-porta* and Queen’s gala dinner

2. Contact time from Tupper and co-authors [63].

In our study the iceberg-like pattern of COVID-19 could be observed, considering the percentages of 0.17% patient deaths, 2.3% hospitalized, 12% asymptomatic, and 85.8% symptomatic [71]. The most frequent symptoms of COVID-19 cases were weakness, fever, and lost smell or/and taste; the severe course of the disease was less frequent in accordance with the average age of the patients [59, 72, 73]. In addition, the medical assistance was low and chronic illness was weakly associated with COVID-19, but cardiovascular diseases, hypertension, and hypothyroidism increased the risk of COVID-19, as indicated in some studies [74, 75]. The presence of sequels remains low, but this is a new disease and follow-up of these patients would be useful.

Considering the risk factors of COVID-19, higher age was associated with the disease, and children and adolescents were less affected [76, 77]; in contrast with other studies [78], sex was not a risk factor. Young patients constituted asymptomatic cases, and their frequency was lower than in other studies [79–81]. The validity of self-reported lifestyle data has been considered good [82], and overweight and obesity were risk factors in line with population-based studies of COVID-19 [83, 84]. In addition, Merzon and co-authors [85] found that vitamin D deficiency was a risk factor of COVID-19 infection and severity. Alcohol consumption was a marginal risk factor, but has not been found in some studies [86, 87]. Upper and middle social classes were associated with the disease, in contrast with other studies [85, 88]. Smoking is a controversial factor because it has been found to be both protective and a risk factor [85–87, 89, 90].

When observing the MGEs, it may be useful to consider that participation in the *Falles* festival was extensive, since the population of Borriana, both adults and children, took part in many cultural, touristic, leisure, and dinner events [35], and many MGEs were held in February and March 2020. These MGEs may be considered as super-spreading events [91, 92]. In this context, several points stand out. First, COVID-19 cases began in January and February 2020, with the onset of symptoms before the MGEs took place. Second, the *pa-i-porta* and the Queen’s gala dinner included dancing, which involved more contact among participants. In these MGEs the presence of participants with symptoms at the same dinner table was a significant risk factor of COVID-19, which is consistent with symptomatic cases being more contagious than asymptomatic cases [10, 93, 94]. Third, indoor MGEs had a high attendance, mainly in building A; considering the building’s capacity and the close proximity among participants, a higher risk of transmission was found [3, 95, 96]. Attendance at the other MGEs was lower and full capacity was not reached. Fourth, several MGEs lasted for more than six hours and ended in the early morning. The average temperature in Borriana in March is 13.4°C, and the average relative humidity is 64% [97]. These weather conditions have been shown to favor SARS-CoV-2 transmission, estimated to range between 5°-15°C and 30–100% of temperature and relative humidity, respectively [98, 99]. Fifth, all the MGEs except the Valencia trip occurred in closed indoor places, building A and theater B; both locations have air conditioning and heating, which are more modern in the case of theater B, and no breakdowns or malfunctions were observed. Finally, airborne transmission [100] may be possible, and our results indicate one quadrant of building A with a lower incidence of COVID-19, but the two quadrants with the highest incidence were not the same in the two events, and these two quadrants were occupied by the *Falles* organizers and guests, who had attended the most MGEs. However, the presence of asymptomatic cases, the variability of the incubation period, and other potential transmission sources make it difficult to confirm this transmission in these MGEs.

A potential causal relationship between MGE attendance and COVID-19 may be considered following the Austin Bradford-Hill criteria [101]; aRR of MGE attendance ranged from moderate to high. It has been suggested that there is a biological plausibility of COVID-19 transmission happening in closed places, where many participants gather in areas that are smaller than 1.83 m^2^ per individual [102]. This is consistent with other COVID-19 outbreaks in MGEs in several countries [21, 24, 42, 103, 104]. The temporal relationship between MGE attendance and COVID-19 was established after the event. A dose-response relationship was demonstrated between MGE attendance, attendance time, and risk of the disease [105]. As an observational study, a retrospective cohort design could estimate the causality relationship more precisely than descriptive studies.

The strengths of our study are as follows. First, it has a population-based design that allows a more integral approach to COVID-19. Second, a representative sample of a population exposed to MGEs was analyzed. Third, the response rate was high. Fourth, a serologic survey was carried out to confirm COVID-19 cases [106, 107] with a highly sensitive and specific technique. Fifth, statistical analysis was adjusted for potential confounding factors. Finally, the sensitivity analysis confirmed the previous results with an improvement in study precision.

The study’s limitations are as follows. First, there was a period of time between exposure at the MGEs and the start of the study. In addition, the impact of the COVID-19 pandemic could have caused some recall and misclassification biases. Second, only the *Falles* population was included in the study. Third, other MGEs were not studied, such as the *Ninot* parade, community dinners, children’s entertainments, and others that may have played a role in COVID-19 transmission in Borriana. Fourth, an estimation of incubation periods was difficult considering the variety of MGE attendance. Fifth, no genetic studies were carried out into the SARS-CoV-2 of positive PCR patients. Sixth, some residual biases may persist despite adjustments. Finally, as a new disease, COVID-19 could have some factors that were not considered in the study.

Some recommendations may be addressed based on the results of the study. First, and with immediate effect, MGEs should be considered as potential triggers of high transmission of SARS-CoV-2. The maintenance, conservation, and inspection of the closed indoor building where these MGEs took place should also be strengthened. We must highlight that MEGs have continued to be held successfully with measures in place to prevent SARS-CoV-2 transmission, suggesting that MGEs could be implemented again with specific prevention measures [108–112]. Second, COVID-19 patients should be monitored to find out about this new disease and its evolution. Third, population-based studies, including serologic surveys, should be carried out to estimate the extent of COVID-19 [113, 114]. Finally, regarding our study, new lines of research could be implemented, including estimating other risk factors of COVID-19 disease, determining serologic variations of antibodies against SARS-CoV-2 in the coming months, following-up patients and potential sequels, and exploring factors associated with the spread of the COVID-19 pandemic [115].

As an epilogue to our study, we are carrying out a prospective cohort study with patients who tested positive for COVID-19, and a second sero-epidemiological study, started in October 2020, to research evolution, sequelae and antibodies against SARS-CoV-2.

## Conclusions

The results of this study suggest the importance of MGEs in COVID-19 transmission that could explain the subsequent COVID-19 outbreak in Borriana. Population-based studies, including serologic COVID-19 surveys, may usefully inform the adoption of preventive measures that help to contain the COVID-19 pandemic.

## Supporting information

S1 File (DOCX)

S1 Data (DTA)

S2 Data (DTA)

S1 Dataset (DOCX)
