# Supplementary material for: “Mass gathering events and COVID-19 transmission in Borriana (Spain): A retrospective cohort study”
Source: PLoS One. 2021 Aug 26;16(8):e0256747. doi: 10.1371/journal.pone.0256747 (PMC8389516; doi:10.1371/journal.pone.0256747)
Supplement: S1 File — (DOCX) [file pone.0256747.s001.docx]

**CONSENTIMIENTO INFORMADO INICIAL**

**(Contacto verbal con la persona)**

**Título del proyecto:**

**“EVENTOS MULTITUDINARIOS Y TRANSMISSION DE LA ENFERMEDAD POR COVID-19”**

Nombre y Apellidos_______________________________________

**Presentación:** Mi nombre es ______________________médico/enfermera del ___________

Estamos realizando un estudio sobre la enfermedad por coronavirus en Borriana para tratar de prevenir esta enfermedad. Por ello solicitamos su cooperación que puede ser de gran valor.

Procedimiento del estudio: En el caso que desee participar, le haremos una serie de preguntas sobre su salud, hábitos de vida, y participación en eventos falleros mediante un cuestionario.

-¿Desea Vd. Participar? Si No.

**Aclaraciones a realizar en la llamada telefónica:**

-La participación en el estudio es voluntaria y en cualquier momento puede abandonar el estudio, sin perjuicio alguno por su parte.

-Podrá recibir información sobre el estudio.

-La información personal que se obtenga de este estudio para la identificación de cada paciente será mantenida en la más estricta confidencialidad.

-Los datos recogidos podrán ser utilizados de forma anónima para la divulgación científica, salvaguardando su intimidad.

**Consentimiento expreso:**

Dadas las características del estudio y que la encuesta se realiza telefónicamente, se sobreentiende que además de responder afirmativamente a la pregunta si desea participar, el hecho de contestar el cuestionario ratifica dicho consentimiento.

Para que la investigación se lleve a cabo con escrupuloso respeto a los principios éticos y no quede duda de la voluntariedad de participación, se identifica el investigador responsable de la recogida de datos.

El interesado/a El investigador que realiza la encuesta

­­­­­­­­­­­­­En__________ a______ de_______2020

**CUESTIONARIO-BORRIANA-1º**

Cuestionario Nº: __________ Fecha: ______________ Entrevistador: __________________

**A. Datos personales**

Nombre y Apellidos:___________________________ Falla: _______________________________

Edad: ____ Sexo: _____ Ocupación: __________________

Domicilio: __________________________ Teléfono:_________ email: ____________________

Peso: _____ Altura: ______ Fumador: Si No Exfumador: Si No No-fumador: Si No

Consume bebidas alcohólicas: Si No

¿Realiza ejercicio físico habitual? Si No ¿Sigue una dieta nutricional? Si No

**B. Participación en eventos falleros**

**Día Viernes 6-Marzo-2020: PAIPORTA FALLERO: 21.30 Llar Fallero**

Participó en este evento: Sí No

¿Recuerda hasta cuando estuvo en el evento?

Solo al principio, hasta la mitad, más de la mitad, hasta el final.

Recuerda la mesa donde estaba: Si No Detallar___________________________________________

Ceno la comida de su falla: Si No Cena propia: Si No

Tartas de concurso: Si No

¿Comió otros alimentos de las mesas?: Si No

¿Recuerda cuánto comió en la cena?: Casi nada, poco, bastante, mucho

¿Llego después de la cena?: Si No

Tomo bebidas alcohólicas: Si No Cantidad: _____ (Indicar cantidad aproximada)

Cerveza Vino Cognac Whisky Ginebra Combinados Cubata

¿Se quedó al baile? Sí No

¿Cuánto bailó? Nada poco bastante mucho

**Día Sábado 7-Marzo-2020: CENA HOMENAJE REINA FALLERA: 22.00 Llar Fallero**

Participó en este evento: Sí No

¿Recuerda hasta cuando estuvo en el evento?

Solo al principio, hasta la mitad, más de la mitad, hasta el final.

Recuerda la mesa donde estaba: Si No Detallar: _________________________________________

Comió cena: Si No Completa: Si No

Entrantes:

Pan-tomate: Si No Embutidos: Si No Ensalada queso-foie: Si No Zamburiñas: Si No.

Primer plato: Solomillo ibérico: Si No

Postre: Crema de chocolate i frambuesa: Si No

Tomo bebidas alcohólicas. Si No Cantidad: ______ (Indicar cantidad aproximada)

Cerveza Vino Cognac Whisky Ginebra Combinados Cubata

¿Recuerda cuánto comió en la cena?: Casi nada, poco, bastante, mucho

**Día Domingo 8-Marzo-2020: Viaje a Valencia “Mascletà” de fallas: Pirotecnia Marti de Borriana.**

Participó en este evento: Sí No

Viajó a Valencia en:

Autobús contratado: Si No Tren: Si No Coche particular: Si No

¿Comió en algún restaurante en Valencia? Si No

¿Recuerda el nombre del restaurante? Si No Detallar________________________________

**Día Martes 10-Marzo-2020: PLEITESIA REINA FALLERA INFANTIL 19.00 Teatro Payá**

Participó en este evento: Sí No

¿Recuerda hasta cuando estuvo en el evento?

Solo al principio, hasta la mitad, más de la mitad, hasta el final.

**Otros eventos falleros:**

-BAILE DE LA 3ª EDAD (Domingo 8: 17.30 Llar fallero): Participó en el evento: Si No

-GALA DEL TEATRO (Domingo 8 19.00 Teatro Payá): Participó en el evento: Si No

-Del 6 al 10 de Marzo:

¿Cuánto dormía cada noche? Muy poco, poco, algo, bastante, mucho

Entre estos días, ¿Cuántas actividades no habituales realizó?

Muy pocas, pocas, algunas, bastantes, muchas.

¿Durante los eventos indicados, recuerda alguna persona con tos o resfriado? Sí No

Detallar el evento _____________________________________

¿Durante estos eventos, piensa que pudo tener algún contacto con una persona positiva al coronavirus?

Piensa que no, poco probable, bastante probable, muy probable.

**C. Situación de salud**

¿Cómo es su salud habitual? mala, regular, buena, muy buena.

¿Sufre alguna enfermedad? Sí No detallar­­­­___________________________________________

¿Toma alguna medicación? Sí No detallar____________________________________________

¿Toma vitaminas habitualmente? Sí No detallar________________________________________

Desde el día 1 de Marzo: ¿Ha tenido alguna enfermedad? Sí No detallar_______________________

¿A partir de Marzo ha tenido síntoma alguno de estos síntomas?:

Tos: Si No Secreción nasal: Si No Dolor de garganta: Si No Fiebre: Si No

Pérdida gusto/olfato: Si No Debilidad: Si No Dolores musculares: Si No

Diarrea: Si No Vómitos: Si No

Cefaleas Si No Febricula Si No Lesiones en la piel: Si No

Otros síntomas: detallar_________________________

Cuantos días duró la enfermedad:______

¿Ha sufrido la enfermedad por coronavirus? Sí No

Consulta médica: Si No Ingreso al Hospital: Si No

¿Podría indicar la fecha de inicio de la enfermedad o de los síntomas indicados? _____

¿Le han efectuado pruebas del coronavirus para saber si ha sufrido la enfermedad?

Sí No detallar_______________________________________________________

Si ha sufrido la enfermedad por coronavirus: ¿Ha pensado como se pudo contagiar?

Si No detallar _________________________________________________________

¿Cómo se encuentra actualmente? Mal, regular, bien, muy bien.

¿Ha presentado alguna secuela después de sufrir la enfermedad por coronavirus? Si No detallar

¿Algún familiar conviviente ha sufrido la enfermedad por coronavirus?

Sí No detallar_________________________________________________________

¿Quiere añadir algún aspecto que piense de interés?

______________________________________________________________________

**D. Observaciones**

**English translation:**

**INITIAL INFORMED CONSENT**

**(Verbal contact with the person)**

**Proyect title:**

**“Mass gathering events and transmission of COVID-19 disease”**

Name and surname _______________________________________

**Presentation:** My name is ______________________physician/nurse from ___________

We are carrying a study on the coronavirus disease in Borriana and the objective is the prevention of this disease. We ask for your cooperation which can be of great value.

Study procedure: If you want to participate, some questions about your health, life style and attending “falleros” (members of fallas) events will be made through a questionnaire.

¿Do you want to participate? Yes No

Clarifications to be made in the phone call

The participation in this study is voluntary, and in at any time you can leave the study, without prejudice on your part.

You can receive information about the study.

The personal information obtained from this study for the identification of each patient

will be kept in the strictest confidentiality.

The data collected will be used anonymously for scientific research, safeguarding your privacy.

**Explicit consent:**

Given the characteristics of the study and that the survey is carried out by telephone, it is understood that in addition to answering affirmatively to the question if you want to participate, the fact of answering the questionnaire ratifies said consent.

So that the research is carried out with scrupulous respect for ethical principles and there is no doubt about the voluntary of participation, the researcher responsible for data collection is identified

The participant the researcher conducting the survey

­­­­In­­­________ date______ of______2020

**QUESTIONNAIRE -BORRIANA-1º**

Questionnaire Nº: __________ Date: ______________ Interviewer __________________

1. **Personal information**

Name and surname:____________________ Falla: _______________________________

Age: ____ Sex: _____ Occupation: __________________

Home: __________________ Telephone:_________ email: ____________________

Weight: ___ Height: ___

Current smoker: Yes No Ex-smoker: Yes No No-smoker: Yes/No

Do you drink alcoholic beverages ?: Yes No

Do you do usual physical exercise ? Yes No ¿Do you follow a nutritional diet? Yes No

**B. Events” falleros” participation**

**Friday March 6, 2020: PAI-PORTA: 21.30 *Fallas* home (building A).**

Do you attended at this event? Yes No

Do you remember the time of attendance at this event?

Less half____ half___ more half____ all the time_______

Do you remember the table where you were? Yes No Details___________________________________________

Do you eat the food of your *falla*? Yes No Own dinner: Yes No

Cake of competition: Yes No

Do you eat other foods from the tables? Yes No

Do you remember how much to ate for dinner? Almost nothing, little, quite, a lot.

Do you come after the dinner? Yes No

Do you drink alcoholic beverages? Yes No

Drink alcoholic beverages? Yes No Amount ____Detail approximate amount

Beer Wine Cognac Whisky Gin drink Mix alcoholics Gin and coca-cola (Cuba libre or Cubata)

Do you stay for the dance? Yes No

How much to you dance? Almost nothing, little, quite, a lot.

**Saturday March 7, 2020: QUEEN GALA DINNER: 22.00 *Fallas* home (building A)**

Do you attended at this event? Yes No

Do you remember the time of attendance at this event?

Less half____ half___ more half____ all the time_______

Do you remember the table where you were? Yes No Details___________________________________________

Do you eat the dinner? Yes No The complete dinner? Yes No

Starters dinner:

Tomato bread: Yes No Pork sausage: Yes No Salad-cheese-foie: Yes No Scallops: Yes No.

First course: Iberian sirloin: Yes No

Dessert: Chocolate cream and raspberry: Yes No

Do you drink alcoholic beverages? Yes No

Drink alcoholic beverages? Yes No Amount ____Detail approximate amount

Beer Wine Cognac Whisky Gin drink Mix alcoholics Gin and coca-cola (Cuba libre or Cubata)

Do you remember how much to ate for dinner? Almost nothing, little, quite, a lot.

**Sunday March 8, 2020: TRIP TO VALENCIA “Mascletà” OF *fallas:* PyrotecHnY “Marti” OF Borriana.**

Do you participate in this event? Yes No

The trip to Valencia was by:

Hired bus: Yes No Train: Yes No Private car: Si No

Do you eat in a restaurant in Valencia? Yes No

Do you remember the name of the restaurant? Yes No

Detail ________________________________

**Tuesday March 10, 2020: PLEITESIA REINA FALLERA INFANTIL 19.00 “Paya” Theatre.**

Do you participate in this event? Yes No

Do you remember the time of attendance at this event?

Less half____ half___ more half____ all the time_______

**Other mass gathering eventos of *Fallas:***

SENIOR’S DANCE (Sunday 8: 17,30 C *Fallas* home (building A):

Do you participate in this event? Yes No

THE THEATRE’S AWARD GALA ((Sunday 8: 17,30 C *Fallas* home (building A):

Do you participate in this event? Yes No

From March 6 to March 10:

How much did you sleep at night?

Very little, little, something, quite, a lot

During these days: How many unusual activities did you do?

Very few, few, some, quite, many

During the indicated events, do you remember someone with a cough or cold? Yes No

Detail the event _____________________________________

During these events, do tou think you may have had contact with a person who is positive for the coronavirus?

Think no, few likely, quite likely, very likely

**C. Health status**

How is your usual health? Very poor poor good excellent

Do you suffer from any disease Yes No

Detail_____________________________________

Do you take any medications? Yes No

Detail_______________________________________

Do you take vitamins regularly? Yes No

Detail________________________________________

From March 1, have you had any illness? Yes No

Detail__________________________________________

From March, have you had some of these symptoms?

Cough; Yes No Coryza: Yes No Sore thoat: Yes No Fever: Yes No

Lost of smell and/or taste: Yes No Weakness: Yes No Diarrhea: Yes No

Vomiting: Yes No Myalgia: Yes No Headache: Yes No Few fever: Yes No

Skin lesions: Yes No

Other symptoms: Detail_________________________

How many days did the disease last? ______

Have you suffered from the coronavirus disease? Yes No

Medical assistance Yes No Hospital admission: Yes No

Could you indicate the date of onset of the disease or the indicated symptoms?

_____________________________________________

Have you been tested to know if you have suffered from the coronavirus disease?

Yes No detail _______________________________________________________

If you have suffered from the coronavirus disease: Have you thought about how you could get it? Yes No detail _________________________________________

-How is your health at the moment?

Very poor, poor, good, very good.

Do you have any sequelae after suffering from the coronavirus disease? Yes No

Detail ______________

Do you have a family member who has suffered from the coronavirus disease? Yes No

Detail _________________________________________________________

Do you want to add any aspect that you consider of interest?

______________________________________________________________________

**D. Observations**
