# Supplementary material for: “Mass gathering events and COVID-19 transmission in Borriana (Spain): A retrospective cohort study”
Source: PLoS One. 2021 Aug 26;16(8):e0256747. doi: 10.1371/journal.pone.0256747 (PMC8389516; doi:10.1371/journal.pone.0256747)
Supplement: S1 Dataset — (DOCX) [file pone.0256747.s004.docx]

STATA® 14 version

Dataset: borriana6661.dta.

Dataset: paiporta661.dta

Title:

“MASS GATHERING EVENTS AND COVID-19 TRANSMISSION IN BORRIANA (SPAIN): A RETROSPECTIVE COHORT STUDY.”

Author`s correspondence addresses

Alberto Arnedo-Pena

C/L’Olivera 5,2-C. Castelló de la Plana 12005 (Spain)

Telephone +34 964 22 96 39 // Mobile +34 622 57 39 79

Email: albertoarnedopena@gmail.com

-----------------------------------------------------------------

Dataset: borriana6661.dta

STATA® 14 version

Variables 132

1. nn: number of the participant

2. age = age (years)

3. sex = sex………………. male=1 female=0

4. loca = municipality: Borriana=1; Alquerias=2; Vall d’Uixo =3; Vila-real=4; Nules=5; Valencia=6; others=7

5. pe = weight (kg)

6. al = height (m)

7. fum = smoking; ………………..No smoking=0; Ex-smoking =1 Current-smoking=2

8. oh = usual alcohol drink: Yes=1 No=0

9. fi = usual physical exercise: Yes=1 No=0

10. nu = follow a nutritional diet: Yes=1 No=0

11. pai = attendance pa-i-porta event : Yes=1 No=0

12. patem = attendance time pai-i-porta Less half=1 half=2 More half=3 All time=4

13. cena = dinner: food dinner from owner =1 food dinner from *Falla* =2

14. ta = cake consumption: Yes=1 No=0

15. otro = other food dinner consumption: Yes=1 No=0

16. cenoh = drink alcohol beverages in pai-i-porta event: quantity of (OH gr).

17. bal = dance in the pai-i-porta event Nothing=0 Few=1 Quite=2 More=3

18. reina = queen gala dinner event Yes=1 No=0

19. retem = attendance time queen gala dinner event

Less half=1 half=2 More half=3 All time=4

20. en1 = tomato bread consumption: Yes=1 No=0

21. en2 = pork sausages consumption: Yes=1 No=0

22. en3 = salad-foie consumption: Yes=1 No=0

23. en4 = scallops consumption: Yes=1 No=0

24. sal = sirloin consumption: Yes=1 No=0

25. cre = chocolate cream consumption: Yes=1 No=0

26. reoh = drink alcohol beverages in queen gala dinner: quantity of (OH gr).

27. via = Valencia trip event: Yes=1 No=0

28. ple20 = Queen offering event attendance Yes=1 No=0

29 bal3 = Senior’s dance event attendance: Yes=1 No=0

30. theat8 = Theater awards event attendance: Yes=1 No=0

31.cont = contact with a COVID-19 case:

No likely=1; Unlikely=2; Quite Likely =3: Very likely=4

32. health = health status before COVID-19 pandemic:

Bad=1; Fair=2; Good=3; Very good=4)

33. disease = chronic disease: Yes=1 No=0

34. med = medication intake: Yes=1 No=0

35. vit = habitual vitamin intake: Yes=1 No=0

36. enf = a disease in the study period Yes=1 No=0

37. tos2= cough: Yes=1 No=0

38. secr = coryza: Yes=1 No=0

39. dolg = sore thoat: Yes=1 No=0

40. fiebre =fever Yes=1 No=0

41. perdida = lost of smell and/or taste: Yes=1 No=0

42. deb = weakness: Yes=1 No=0

43. dia = diarrhea: Yes=1 No=0

44.vom = vomiting: Yes=1 No=0

45. atenmed = medical assistance: Yes=1 No=0

46. hoy = actual health status:

Very poor=1; Poor=2; Good=3; Very good=4)

47. famcon = a family with COVID-19 disease: Yes=1 No=0

48. falla = number of each falla: La Merce = 1; Barri Valencia = 2; La Vila = 3; Escorridor = 4; Onda = 5; Ravalera = 6;Sant Blay = 7; La Mota = 8; Caçador =9 España=10; Chicharro=11; Club 53 =12; La Bosca =13; Club Ortega = 14; D.Bosco=15 ; Quarts de Calatrava = 16; Sant Josep = 17; Sant Jaime =18; Cardenal Tarancón =19:

*Fallas* organization team (local board ) and the guests of queen gala dinner = 20;

Food-handlers: pai-i-porta, queen gala diner, and senior’s dance events = 21.

49. eventos = number of events: 0,1,2,3,4,5

50. mial = myalgia: Yes=1 No=0

51.cefa = headache: Yes=1 No=0

52.febrícula= few fever: Yes=1 No=0

53. class = social class

From occupation:

Group I y II: professional, managerial and technical occupations; class =1

Group III-VI: skilled, non-manual or manual; partly-skilled; unskilled occupations;

class = 0

54. derm = dermatologic lesions Yes=1 No=0

55.hospital = hospitalization Yes=1 No=0

56. solo baile = only dance in the pai-i-porta event:

Nothing=1; Few=2; Quite=3; More=1

57. PCR = positive polymerase chain reaction test: Yes=1 No=0

58. ant = positive serology SARS-CoV-2 serology; Yes=1 No=0

59. igm= positive IgM SARS-CoV-2 serology; Yes=1 No=0

60. igg = positive IgG SARS-CoV-2 serology; Yes=1 No=0

61. disnea = dypnea Yes=1 No=0

62. dias = duration COVID-19 disease: quantitative (days)

63. neumonia = pneumonia; Yes=1 No=0

64. mesa = number dinner table queen gala dinner :0-40

65. newigg = positive IgG SARS-CoV-2 serology Yes=1 No=0 in the study carried out June, 2020.

66.postcobi = sequelae post-COVID-19:

Weakness =1; dypnea = 2; asthma increase = 3; lost smell and/or taste = 5; digestive illness = 6; lost weight =7; dermatologic lesions =10; sore throat or cough =11; afraid illness =13; dizziness=30

67. caseend = case of COVID-19 Yes=1 No=0: FINAL CASE.

68. mayores = groups of age (years)

0-4 =1; 5-14 = 2; 15-24 = 3; 25-34 = 4; 35-44 = 5; 45-64 = 6; 65 and above = 7

69. BMI= body mass index: quantitative (Kg/m^2^)

70. pesomio= BMI in range:

<18.5 = 0; 18.5-24.9 =1; 25.0-29.9 = 2; ≥ 30.0 =3:

71.obi = obesity BMI>≥ 30.0 Yes=1 No=0

72. ee1 = number of events attendance = 0 Yes=1 No=0

73. ee2 = number of events attendance =1 Yes=1 No=0

74. ee3 = number of events attendance =2 Yes=1 No=0

75. ee4 = number of events attendance =3 Yes=1 No=0

76. ee5 = number of events attendance = 4 Yes=1 No=0

77. ee6 = number of events attendance = 5 Yes=1 No=0

78. asintomatic = COVID-19 case without symptoms Yes=1 No=0

79. labora = COVID-19 case with a confirmation test Yes=1 No=0

80. sample2= No-cases and COVID-19 cases onset: March 6^th^ and 31^st^ Yes=1;

COVID-19 cases onset before Martch 6^th^ =2;

COVID-19 cases onset after March 31^st^ = 3

81.before = COVID-19 cases onset before Martch 6^th^ Yes=1; No=0

82. smoker22 = No smoking=0; Ex-smoking =1 Current-smoking=2

83. tab1 = No smoking Yes=1; No=0

84. tab2 = Ex-smoking Yes=1; No=0

85. tab3= Current-smoking Yes=1; No=0

86. cc1 = Contact with a positive coronavirus case: No likely: Yes=1; No=0

87. cc2 = Contact with a positive coronavirus case: Unlikely Yes=1; No=0

88. cc3 = Contact with a coronavirus case : Quite likey Yes=1; No=0

89. cc4 = Contact with a coronavirus case:Very likey Yes=1; No=0

90. oo1 = pesomio: BMI <18.5 Yes=1; No=0

91. oo2 = pesomio: BMI 18.5-24.9 Yes=1; No=0

92. oo3 = pesomio: BMI 25.0-29.9 Yes=1; No=0

93. oo4 = pesomio: BMI ≥ 30.0 Yes=1; No=0

94. tt1 = attendance time pai-i-porta Less half Yes=1; No=0

95. tt2 = attendance time pai-i-porta Half Yes=1; No=0

96. tt3 = attendance time pai-i-porta More half Yes=1; No=0

97. tt4 = attendance time pai-i-porta All time Yes=1; No=0

98. cardio = cardiovascular disease Yes=1; No=0

99. diabe = diabetes mellitus Yes=1; No=0

100. asma = asthma Yes=1; No=0

101. rinitis = rhinitis Yes=1; No=0

102. hipotiro = hypothyroidism Yes=1 No=0

103. neuro = neurological diseases Yes=1 No=0

104. digestiva = digestive diseases Yes=1 No=0

105. respir = respiratory diseases Yes=1 No=0

106. tension = hypertension Yes=1 No=0

107. psique = mental illnessYes=1 No=0

108. rr1 = attendance time queen gala dinner Less half Yes=1 No=0

109. rr2 = attendance time queen gala dinner Half Yes=1 No=0

110. rr3== attendance time queen gala dinner More half Yes=1 No=0

111. rr4 = attendance time queen gala dinner All time Yes=1 No=0

112.bb1= dance in the pai-i-porta event Nothing Yes=1 No=0

113.bb2 = dance in the pai-i-porta event Few Yes=1 No=0

114.bb3 dance in the pai-i-porta event Quite Yes=1 No=0

115.bb4 = dance in the pai-i-porta event More Yes=1 No=0

116. carrier = a participant in the queen gala event and reporting COVID-19 disease and the onset of symptoms between February 27^th^ and March 7^th^. Yes=1 No=0

117.uuu1: cuadrant1 building A Yes=1 No=0

118. uuu2 : cuadrant 2 building A Yes=1 No=0

119.uuu3 : cuadrant 3 building A Yes=1 No=0

120.uuu4 : cuadrant 4 building A Yes=1 No=0

121. mm1 mayores = groups of age 0-4 years Yes=1 No=0

122. mm2 = groups of age 5-14 years Yes=1 No=0

123. mm3 = groups of age 15-24 years Yes=1 No=0

124. mm4 = groups of age 25-34 years Yes=1 No=0

125.mm5 = groups of age 35-44 years Yes=1 No=0

126. mm6 = groups of age 45-64 years Yes=1 No=0

127. mm7 = groups of age 65 and above years Yes=1 No=0

128. cuadrant2 = the division of building A in four quadrants

-----------------------Stage----------------------

| 1 Upper left | 2 Upper right * |
| --- | --- |
| 3 Lower left ** | 4 Lower right * |

*Emergency Exit **Entrance

129. sdate = data of the onset COVID-19 cases.

130.ocup = occupations

professional, managerial =1; technical occupations =2

skilled, non-manual =3; skilled manual =4 ; partly-skilled =5; unskilled =6;

students= 10; paro = 15; retired =20; house keeper =30

131. ohpai= Drink alcohol beverages in pai-i-porta event Yes=1 No=0

132. ohreina = Drink alcohol beverages in queen gala dinner Yes=1 No=0

Dataset: paiporta6661.dta

STATA® 14 version

Variables 9

Ratepai = rate of COVID-19 case per each *falla* during pai-i-porta event.

carrie2= a participant in the pa-i-porta and reporting COVID-19 disease and the onset of symptoms between February 26^th^ and March 6^th^ Yes=1 No=0

Sex = Percent of female in each *falla*

Age = median of age in each *falla*

Cuadrant= the division of building A in four quadrants

-----------------------Stage----------------------

| 1 Upper left | 2 Upper right * |
| --- | --- |
| 3 Lower left ** | 4 Lower right * |

*Emergency Exit **Entrance

Eventsmean =mean MGES attendance

Paitem = mean of attendance time

nn= number

fallanumber = = number of each falla: La Merce = 1; Barri Valencia = 2; La Vila = 3; Escorridor = 4; Onda = 5; Ravalera = 6;Sant Blay = 7; La Mota = 8; Caçador =9 España=10; Chicharro=11; Club 53 =12; La Bosca =13; Club Ortega = 14; D.Bosco=15 ; Quarts de Calatrava = 16; Sant Josep = 17; Sant Jaime =18; Cardenal Tarancón =19:

*Fallas* organization team (local board) and the guests of queen gala dinner = 20
